# Brain temperature and its fundamental properties: a review for clinical neuroscientists

**DOI:** 10.3389/fnins.2014.00307

**Published:** 2014-10-08

**Authors:** Huan Wang, Bonnie Wang, Kieran P. Normoyle, Kevin Jackson, Kevin Spitler, Matthew F. Sharrock, Claire M. Miller, Catherine Best, Daniel Llano, Rose Du

**Affiliations:** ^1^Department of Neurosurgery, Carle Foundation Hospital, University of Illinois College of Medicine at Urbana-ChampaignUrbana, IL, USA; ^2^Thermal Neuroscience Laboratory, Beckman Institute, University of Illinois at Urbana-ChampaignUrbana, IL, USA; ^3^Department of Internal Medicine, Carle Foundation Hospital, University of Illinois College of Medicine at Urbana-ChampaignUrbana, IL, USA; ^4^Department of Internal Medicine, College of Medicine at Urbana-Champaign, University of IllinoisChampaign, Urbana, IL, USA; ^5^Department of Molecular and Integrative Physiology, University of Illinois College of Medicine at Urbana-ChampaignUrbana, IL, USA; ^6^Neuroscience Program, University of Illinois at Urbana-ChampaignUrbana, IL, USA; ^7^Molecular and Cellular Biology, University of Illinois at Urbana-ChampaignUrbana, IL, USA; ^8^Department of Neurosurgery, Brigham and Women's Hospital, Harvard Medical SchoolBoston, MA, USA

**Keywords:** brain, temperature, cerebral blood flow, hypothermia, hyperthermia

## Abstract

Brain temperature, as an independent therapeutic target variable, has received increasingly intense clinical attention. To date, brain hypothermia represents the most potent neuroprotectant in laboratory studies. Although the impact of brain temperature is prevalent in a number of common human diseases including: head trauma, stroke, multiple sclerosis, epilepsy, mood disorders, headaches, and neurodegenerative disorders, it is evident and well recognized that the therapeutic application of induced hypothermia is limited to a few highly selected clinical conditions such as cardiac arrest and hypoxic ischemic neonatal encephalopathy. Efforts to understand the fundamental aspects of brain temperature regulation are therefore critical for the development of safe, effective, and pragmatic clinical treatments for patients with brain injuries. Although centrally-mediated mechanisms to maintain a stable body temperature are relatively well established, very little is clinically known about brain temperature's spatial and temporal distribution, its physiological and pathological fluctuations, and the mechanism underlying brain thermal homeostasis. The human brain, a metabolically “expensive” organ with intense heat production, is sensitive to fluctuations in temperature with regards to its functional activity and energy efficiency. In this review, we discuss several critical aspects concerning the fundamental properties of brain temperature from a clinical perspective.

## Introduction

The brain comprises only 2% of human body mass, yet accounts for 25% of the body's total glucose utilization and 20% of oxygen consumption (Squire, 2012). It is a metabolically “demanding” organ with intense heat production (Lamanna et al., 1980; Yablonskiy et al., 2000; Howarth et al., 2012). Almost all cerebral processes are sensitive to temperature fluctuations (Guatteo et al., 2005; Weimer and Hanke, 2005; Fohlmeister et al., 2010; Kiyatkin, 2010). With its energy expenditure efficiency being highly temperature-dependent (Yu et al., 2012), the brain's thermal regulatory capacity may define its anatomical and physiological architecture and constrain its processing capacity.

As one of the key homeostatic parameters, temperature fluctuations intrinsically modulate behavioral changes and reflexively generate autonomic responses (Craig et al., 2000). As endotherms, humans regulate body temperature quite closely to a basal mean that varies little in the absence of pathology. It might therefore be surprising that thermal differences exist at all in the brain, yet the presence of such differences indicate it would be more surprising if they were not functional in some way.

To date, preclinical and clinical data strongly indicate that a destructive relationship exists between brain temperature elevation and cerebral injuries; conversely, brain hypothermia, with its broader, pleiotropic effects, represents the most potent neuroprotectant in laboratory studies (Dietrich et al., 2009). Hypothermia has been shown to protect against excitotoxicity, specifically the glutamate- and dopamine-associated cerebral cytotoxicity of global ischemia (Dietrich et al., 2009). Injury models show increased tissue preservation with hypothermia following injury, even after injury processes become established at basal temperatures (Maybhate et al., 2012).

Brain hypothermia has well-established therapeutic roles in selected clinical conditions, including anoxic brain injury due to cardiac arrest (Bernard et al., 2002; Hypothermia after Cardiac Arrest Study Group, 2002; Nielsen et al., 2013) and hypoxic ischemic neonatal encephalopathy (Gluckman et al., 2005; Shankaran et al., 2005). However, efforts to expand the use of therapeutic hypothermia in other major clinical conditions, such as stroke and head trauma, have had mixed results at best (Clifton et al., 2001, 2011; O'Collins et al., 2006; Hutchison et al., 2008). Recent clinical evidence concerning patients suffering cardiac arrest suggests that cooling during reperfusion is key to limiting destructive physiological cascades that result in cellular injury (Kim et al., 2013). This result is exciting because one of the major limitations of cardiopulmonary resuscitation is the cellular injury associated with reactive oxygen species which occurs on return of spontaneous circulation (Lucchesi, 1990; Neumar et al., 2008).

Avoiding this damage at the cellular level through cooling at the systemic level is one approach that has been attempted and is discussed below. Perhaps a key missing ingredient is a fundamental understanding of temperature dynamics in the brain and the *interactions* between temperature, cerebral blood flow (CBF), regional brain activity and neuronal viability. With a focus on clinical relevance, efforts are warranted to first synthesize what is known about brain temperature regulation, and then propose key research questions to pragmatically develop effective therapeutic strategies.

In this paper, we review the state of our present knowledge concerning brain temperature. Typically, global brain temperature readings assessed in resting clinical patients are congruent with patient body temperature (brain 36.9 ± 0.4°C, rectal 36.9 ± 0.6°C) (Soukup et al., 2002); differences are noted when brain regions are assessed individually. Because denaturation of lipid and protein begins at approximately 45°C and voltage-gated channels exhibit non-Arrhenius behavior at temperatures below 10°C (Collins and Rojas, 1982; He, 2011), this paper will not discuss the extreme temperature ranges.

## Overview

### Historical perspective

Attempts to understand and measure the specific thermal properties of neurological systems began in the mid-19th century. Major advancements proceeded in-stride with improvements in the sophistication of thermal recording equipment. In 1848, the physicist and physiologist Herman von Helmholtz undertook experiments to be the first to measure both the conduction velocity of the action potential, and the heat produced by it in frog motor neuron. However, the equipment of the day did not possess the necessary amplification to characterize the thermal phenomena (Helmholtz, 1848). In 1892, William James wrote “brain activity seems accompanied by a local disengagement of heat.” This was based on Moritz Schiff's 1860s studies of brain temperature in the awake dog, where a 1°C temperature rise was recorded following the presentation of meat (James, 1892). In 1904 and 1908, Walther Nernst demonstrated that the potential across the neural membrane was dependent upon both ionic concentrations and temperature, and discovered the threshold for stimulation of the action potential in the frog sciatic nerve. The first successful experiments to quantify heat production from a train of neural impulses were performed in crustaceans and showed an average increase of 2 μ°C per impulse (Hill, 1929; Feng, 1936).

With improved thermal recording equipment, measurements in the brain of cats revealed several key features: (1) an approximately 1°C gradient exists between the cooler cortical regions and warmer basal regions; (2) the depth of anesthesia correlates with the lowering of brain temperature; (3) brain temperature can be reduced with extracranial cooling; (4) a rise and fall in temperature occurs with sleep and arousal; and (5) a rise of temperature in neuronal pathways is associated with sensory stimuli on an extended time course beyond the duration of the stimulus (Serota and Gerard, 1938; Serota, 1939b).

As knowledge of the mechanisms of the action potential grew, so did the understanding of the temperature dependence of the neural impulse. Hodgkin and Huxley recognized the temperature dependence of the action potential form and the ionic gating parameters by which the impulse is generated (Hodgkin and Huxley, 1952; Huxley, 1957). In 1958, again with the introduction of superior signal amplification and thermopile equipment, the first recordings of heat production from a single neuronal impulse were recorded in the crab (Abbott et al., 1958). These results showed a biphasic response with heat production 80% greater than previously estimated and absorption of heat creating the averaged 2 μ°C signal. This production and absorption was found to be temporally correlated with the depolarization and repolarization of the neural membrane during the action potential (Ritchie, 1973).

In 1966, with an early understanding of the impact of the vascular system on brain temperature, Delgado and Hanai produced temperature changes in the ipsilateral orbital cortex by heating and cooling the exposed carotid artery using irrigation with Ringer's solution (Delgado and Hanai, 1966a). The posterior cruciate gyrus, however, was not similarly cooled. The authors remarked that this was due to “the considerable circulatory independence of these regions.” Their results also confirmed the heterogeneity of intracerebral temperatures, in contrast to the claims of several early investigators (Rampone and Shirasu, 1964; Kawamura and Sawyer, 1965b). Several studies have confirmed temperature gradients between different brain regions in large and small animal studies (Delgado and Hanai, 1966a; Mcelligott and Melzack, 1967b; Hayward and Baker, 1968a; Andersen and Moser, 1995; Moser and Mathiesen, 1996; Thornton, 2003). It was assumed that greater insulation and a warm blood supply generated these gradients.

Interestingly, direct arterial temperature measurements demonstrate that the blood supply is typically cooler than the surrounding basal brain parenchyma (Kiyatkin et al., 2002). Alternative explanations for the observed intracerebral temperature gradients include increased basal rates of neuronal activation and expression of mitochondrial uncoupling proteins (UCPs) in greater quantities in ventral brain regions (Horvath et al., 1999).

### General overview

In general, core brain temperature is higher than body temperature but correlates well with body temperature. Fluctuating in both physiological and pathological conditions, brain temperature itself largely depends on the summed effects of the following principle variables: brain metabolism, CBF and volume, and blood temperature (Hayward and Baker, 1969). Temperature changes of 1°C or less can result in functional alterations in various areas of the nervous system (Brooks, 1983), indicating the high thermal sensitivity of the brain. The significance of thermal impact on several principal neurophysiological properties, such as resting potential, action potential, nerve conduction velocity, and synaptic transmission, is well established (Katz and Miledi, 1965; Brooks, 1983; Thompson et al., 1985; Volgushev et al., 2000; Xie et al., 2000; Rosen, 2001; Tryba and Ramirez, 2004; Lee et al., 2005). The temperature-dependent nature of cerebral functional activity has also been well reported. For example, impairment of memory encoding starts at a body temperature of 36.7°C and progresses to the point that 70% of information normally retained is lost at approximately 34–35°C (Coleshaw et al., 1983).

The brain has a relatively high average Van't Hoff Q_10_ coefficient (the temperature coefficient that defines the change of a chemical reaction rate as a consequence of increasing the temperature by 10°C), on the order of 2.3 in the physiological temperature range (Swan, 1974; Michenfelder and Milde, 1991); however, cells in many cerebral structures demonstrate even higher temperature sensitivity. For example, within the physiological range (34–39°C), murine substantia nigra dopamine neurons *in vitro* (Guatteo et al., 2005) increase their discharge rate with warming (*Q*_10_ = 3.7). Below physiological range (34–29°C), their discharge rate dramatically decreases (*Q*_10_ = 8.5). Temperature intrinsically affects brain metabolic rate as well as affinity of hemoglobin for oxygen (Guyton and Hall, 2006). Neocortical network activity and cellular properties significantly change with temperature in a murine model (Kalmbach and Waters, 2012).

The dynamics of brain temperature fluctuations in humans have not been well characterized. Physiological fluctuations of 1–3°C in brain temperature have been extensively reported in various animal models (Serota, 1938, 1939a; Hamilton, 1963; Hammel et al., 1963; Jackson and Hammel, 1963; Kawamura and Sawyer, 1965a; Hemingway et al., 1966; Delgado and Hanai, 1966b; Hayward and Baker, 1968b; Reite and Pegram, 1968; Kovalzon, 1973; Kiyatkin et al., 2002). Brain temperature fluctuations of this magnitude dramatically change neural function at multiple levels. Action potential generation, trans-membrane ionic transport, and passive membrane properties are altered. Additionally, terminal transmitter release and pre-synaptic uptake processes are perturbed (Katz and Miledi, 1965; Brooks, 1983; Thompson et al., 1985; Volgushev et al., 2000; Xie et al., 2000; Rosen, 2001; Tryba and Ramirez, 2004; Lee et al., 2005).

Emerging research supports the hypothesis that brain temperature alone may act as an active and dynamic variable, capable of regulating or driving brain activity and function (Long and Fee, 2008; Kiyatkin, 2010; Andalman et al., 2011; Aronov and Fee, 2012). However, definitive clinical and laboratory evidence remains to be elucidated. Physiological brain temperature fluctuations in various experimental system as well as clinical populations are typically within a 3°C range (Serota, 1938, 1939a; Mcelligott and Melzack, 1967a; Hayward and Baker, 1969; Sedunova, 1992; Mellergard, 1995; Hirashima et al., 1998; Rossi et al., 2001; Kiyatkin et al., 2002; Wang et al., 2004; Mitchell et al., 2006; Lust et al., 2007; Maloney et al., 2007; Zhu et al., 2009). Surgically exposed cerebral cortex can readily have temperatures 5–10°C below core body temperatures (Gorbach et al., 2003; Kalmbach and Waters, 2012). Therefore, if there is, indeed, an intrinsic and regionally-specific cerebral thermal regulatory mechanism, such a mechanism is likely to operate only within this physiological range.

### Thermal gradients and intracerebral temperature fluctuations

Extensive animal and human data have conclusively established that core brain temperature is generally higher than body temperature, but correlates well with body temperature (Serota, 1938, 1939a; Mcelligott and Melzack, 1967a; Hayward and Baker, 1969; Sedunova, 1992; Mellergard, 1995; Hirashima et al., 1998; Rossi et al., 2001; Kiyatkin et al., 2002; Wang et al., 2004; Mitchell et al., 2006; Lust et al., 2007; Maloney et al., 2007; Zhu et al., 2009). At rest, the human brain has an estimated metabolic rate of 3–3.5 mL O_2_ (100 g cerebral tissue)^−1^ min^−1^ with a corresponding cerebral heat production of approximately 0.6 jg^−1^ min^−1^ (Lassen, 1985; Madsen et al., 1993). Because perfusing blood clears the metabolic heat produced in the brain, the thermal gradient is from the brain (heat source with higher temperatures) to the blood (heat sink with lower temperatures). At rest, cerebral heat balance is established with a jugular-venous-to-arterial temperature difference (v-aD_temp_) of approximately 0.3°C (Yablonskiy et al., 2000; Nybo et al., 2002b).

Studies concerning brain temperature in animals and humans demonstrate results that seem to vary with the method of temperature measurement (thermistor, infrared camera, or magnetic resonance, etc.), the type of stimulus, animal preparation (anesthetized, restrained, or freely moving, etc.), species, age and size of the animal. For example, investigation of brain thermal response to various stimuli by introducing thermistors into the brain differs significantly from studies using infrared imaging. Infrared waves are strongly absorbed by water. A thin layer of fluid (100 um) completely shields the infrared radiation (Schlessinger and Spiro, 1995). Therefore, infrared imaging enables direct thermovision of the brain cortex but is suboptimal for subcortical structures. Due to ready thermal exchange with the ambient air, surgically exposed brain cortex has significantly sub-physiological baseline temperatures (Gorbach et al., 2003; Kalmbach and Waters, 2012). In addition, a thin layer of cerebrospinal fluid (CSF) circulates in the subarachnoid space in between the arachnoid and pia layers that typically covers the exposed cortex. Therefore, the observed thermal vision may underestimate the true magnitude of the cortical thermal response to various stimuli. However, the insertion of thermocouples results in focal tissue injury. This may modify the anatomical arrangement and physiological function of the adjacent neurovascular units and potentially compromise local heat dissipation. Therefore, the data obtained using thermocouples may overestimate the true magnitude of the brain thermal response to various stimuli.

### Animal data overview

Awake or large anesthetized animals have a positive brain-body temperature gradient (brain temperature > body core temperature). It has been widely demonstrated in monkeys, dogs, rabbits, cats, and sheep, that the center of cerebral hemispheres (internal capsule, basal ganglia, and the subcortical white matter in the corona radiata) and the midbrain reticular formation are the hottest parts of the brain, 0.5–0.6°C warmer than the blood (Hayward and Baker, 1969). In unrestrained baboons, using implanted thermometric data loggers, temperatures measured directly in the hypothalamus exceeded the blood temperatures by 0.5°C (Maloney et al., 2007). A positive brain-body temperature gradient was also reported in horses (Mitchell et al., 2006), pronghorn antelopes (Lust et al., 2007), awake cats (Hamilton, 1963; Mcelligott and Melzack, 1967a), freely moving rats (Kiyatkin et al., 2002), and un-anesthetized mice (Sedunova, 1992). As a central nervous system (CNS) thermal buffer, CSF of the basal subarachnoid space in primates was reported to have temperatures at the level of arterial blood temperature (Hayward and Baker, 1968b).

In contrast to awake or large anesthetized animals, small anesthetized animals have a negative brain-body temperature gradient (brain temperature < body temperature). Small animals have a high brain-surface-to-volume ratio to induce significant thermal interactions with the environment. In anesthetized states, possibly due to the suppression of metabolic heat production by the anesthetic agent as well as effective heat exchange with the environment through the head, a negative brain-body temperature gradient has been observed in rats, cats, and miniature pigs (Serota, 1938; Mcelligott and Melzack, 1967a; Lamanna et al., 1989; Laptook et al., 2001).

**Figure 1:**
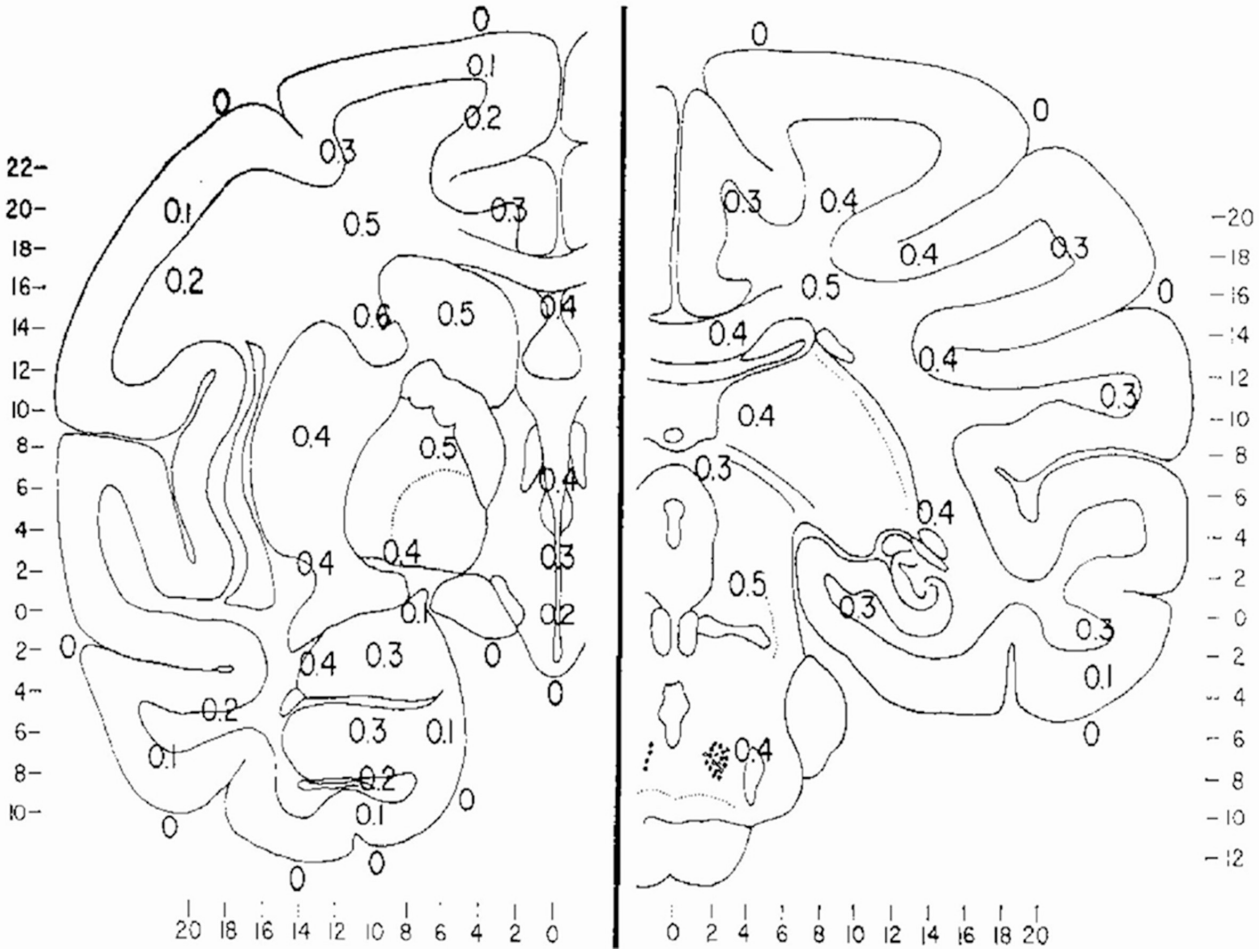
**Thermal map of the brain**. The results of measurements of the temperature difference between arterial blood and 100 brain and subarachnoid sites in 16 monkeys during 347 experiments. Values expressed are T_i_–T_a_ temperature of intracranial site minus the temperature of the aortic arterial blood measured simultaneously, and are all positive values). The major regions which have been studied in the primate brain have been placed on two representative frontal sections, A, frontal 14.5 and B, front 0.3. Values are expressed to the nearest 0.1 C. Reprinted with permission from The American Journal of Physiology, Hayward and Baker (1969).

Cerebral structures appear to have their own basal temperatures, with the temperature differences between various brain regions remaining relatively constant (Kiyatkin et al., 2002). A thermal gradient of 1.4°C between the hypothalamus and the cortex was reported in free moving cats as early as 1938 (Serota, 1938, 1939a). From the results of temperature measurements between arterial blood and 100 brain and subarachnoid sites in 16 monkeys during 347 experiments, Hayward et al. (Hayward and Baker, 1968b) constructed a thermal map of the primate brain (Figure 1). Significant background inter-hemispheric thermal asymmetries have also been reported. For example, the left hemisphere temperature in rats under local anesthesia was noted to be 0.5–0.8°C higher than the right (Shevelev, 1998).

Brain temperature fluctuations related to sleep, arousal, sensory stimulation, and environmental challenges have been reported in monkeys (Hamilton, 1963; Hayward and Baker, 1968b; Reite and Pegram, 1968), rabbits (Kawamura and Sawyer, 1965a), cats (Serota, 1938, 1939a; Delgado and Hanai, 1966b), dogs (Hammel et al., 1963; Jackson and Hammel, 1963), sheep (Hemingway et al., 1966), and rodents (Kovalzon, 1973; Sullivan et al., 1988; Kiyatkin et al., 2002; Trubel et al., 2006). The electrophysiological correlates of spatial learning and memory have a strong relationship with temperature (Erickson et al., 1996). In the awake freely moving rats, temperature in hippocampus and piriform cortex can decrease 0.5–36.5°C over a 1 h period of sleep and quiet wakefulness, and then increase 1.5–38°C when the rat is actively exploring (Andersen and Moser, 1995).

**Figure 2:**
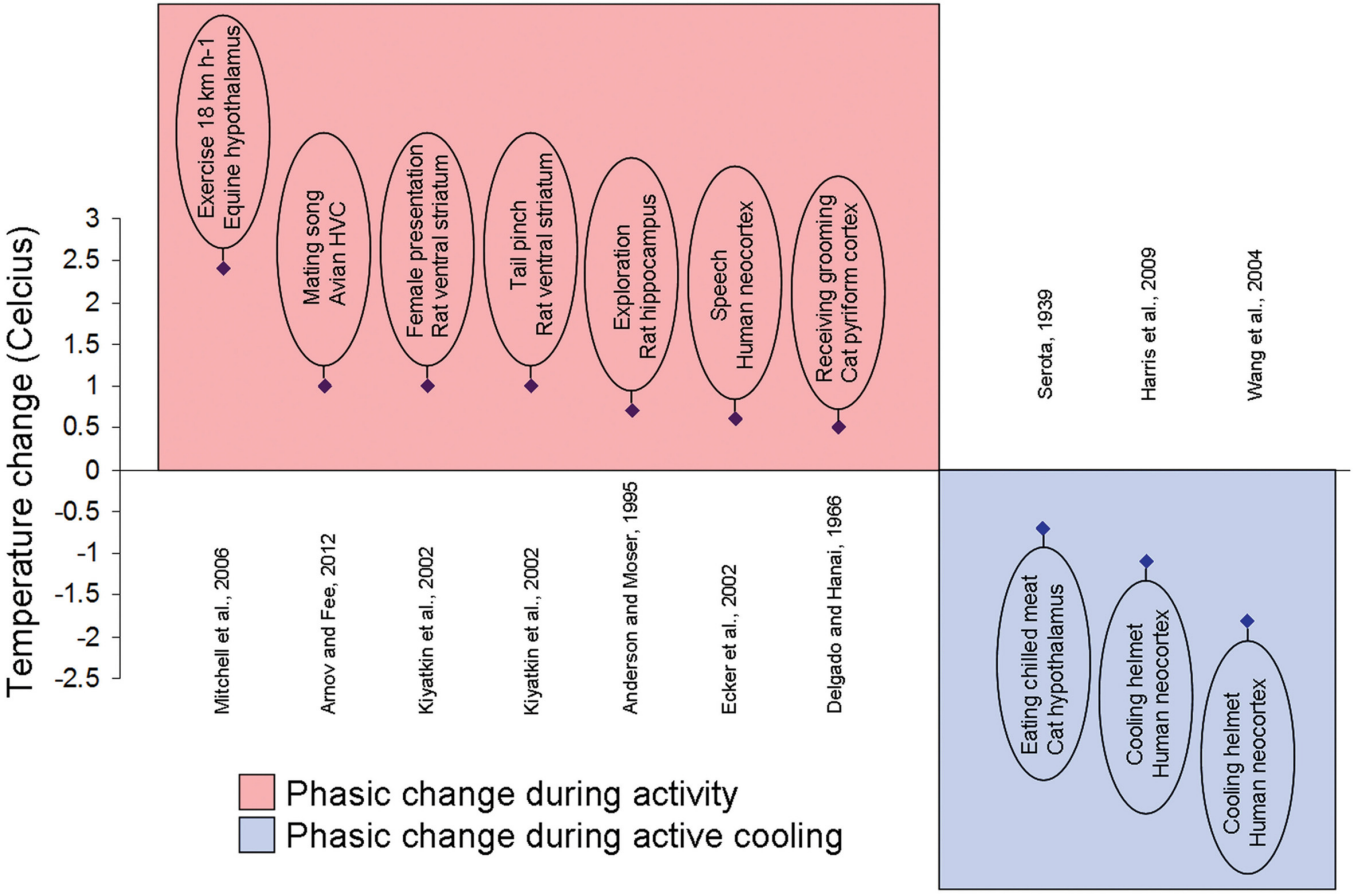
**Local brain temperature change during activity and active cooling**. Example studies documenting localized phasic temperature changes after an increase in activity or a stimulus presentation (plotted over red background) or to external cooling (plotted over blue background). Brief experiment description, species, and brain area written inside oval and reference displayed on opposite side of abscissa.

The temperature fluctuations appear small in anesthetized states but can be quite significant (1–3°C) in freely moving animals (Figure 2). The sexual arousal-related temperature increase in the nucleus accumbens (NAcc) of approximately 1.8°C in the rat was reported by Kiyatkin and Mitchum (2003). Kiyatkin and Brown (2004) and Kiyatkin and Bae (2008) also demonstrated that the magnitude of such fluctuations appeared to depend on the pre-stimulus basal temperature values. In rats, when basal temperatures were low, brain temperature increase was large. However, the thermal response became gradually weaker when basal temperatures were high.

Experimental stimuli such as feeding, social interaction, and tail-pinch induced rapid, unique, and relatively long lasting brain temperature elevations, occurring significantly faster and with greater magnitude than the corresponding temperature elevations in arterial blood (Kiyatkin et al., 2002). The observations that regional brain temperatures rise more quickly and to a larger extent than do arterial blood temperatures suggest that local cerebral heat production may be the primary cause of such functional brain hyperthermia. In addition, these regional brain temperature fluctuations demonstrate structural specificity. For example, in rats, temperature increases in cerebellum were more delayed and prolonged following each stimulus (Kiyatkin et al., 2002). The local thermal responses to certain experimental stimuli also showed a clear habituation pattern in rats. For example, the thermal responses to sound stimuli disappeared by the fifth session (Kiyatkin et al., 2002).

Recent work in songbirds suggests that brain temperature may be an important dynamic variable that actively modulates the function and output of neural circuits. For example, direct and focal brain temperature manipulations have been shown to induce changes in tempo of a similar magnitude (Long and Fee, 2008; Andalman et al., 2011). Brain temperature changes recorded in freely-behaving male finches explained nearly all the variance in song tempo related to the presentation of a female bird as well as to the time of day (Aronov and Fee, 2012). These studies suggest a potential causal effect of temperature on song tempo.

Likewise, *in vivo* mammalian experiments have demonstrated that neuronal activity is a function of temperature (Kalmbach and Waters, 2012). In the anesthetized rat, barrel cortex pyramidal neurons have hyperpolarized membrane potentials at physiological temperature (36°C), while the resting membrane potential is relatively depolarized at cooler temperatures (28°C) (Kalmbach and Waters, 2012). Similarly, the neural network properties of barrel cortex have been investigated in the anesthetized rat, and extrapolation from this study suggests that physiological temperatures place cortical neuron in the down state, in which neurons are more likely to yield action potentials to sensory stimuli (Sachdev et al., 2004). In contrast, cooling the cortex shifts cortical neuron to the up state, known to reduce cortical excitability in barrel cortex (Sachdev et al., 2004). This synthesis of the literature remains to be formally tested, but is expected to be part of the neurophysiological underpinning of the noted neuroprotective state of hypothermia.

Selective cooling of the cortex has been achieved in the primate with cooling probes chronically-implanted over the dura (Fukuda et al., 1987). This method allowed significant localized cooling from approximately 38 to 18°C while permitting performance of an over-learned operant task, although error rate increased by a factor of 10 (Fukuda et al., 1987). Overall, single unit recordings demonstrated reversible decrements in firing rate to preferred stimuli (Fukuda et al., 1987). This study has validated a method to chronically, yet reversibly access and cool the basal surface of primate brain without damaging neural tissue. This portion of the brain is known to contain structures exquisitely sensitive to neurotrauma including the CA1 field of the hippocampus (Nakada et al., 2005; Greenfield et al., 2008).

### Human data overview

On average, deep brain temperature is less than 1°C higher than body temperature in humans, unless cerebral injury is severe enough to significantly disrupt the brain-body temperature regulation (Soukup et al., 2002). Theoretically, the maximal brain temperature elevation over blood temperature, under physiological conditions, would be approximately 0.9°C for a typical hematocrit level of 40% (Yablonskiy et al., 2000), and both the magnitude and direction of the difference can be temperature dependent (Nybo et al., 2002b; Smith et al., 2011). The temperature gap may become more accentuated at higher body temperatures and diminish or even reverse in its relationship at lower body temperatures.

Brain temperature in head trauma patients, directly measured in the frontal white matter at 0.8 cm below the cortical surface, was on average 0.22°C above body temperature (Wang et al., 2004). CSF temperature, measured directly in the frontal horn of the lateral ventricle (4–5 cm below the brain surface), was on average 0.3–0.9°C above body temperature (Hirashima et al., 1998; Rossi et al., 2001). Consistent with extensive animal data (see above), human brain temperatures measured at various cerebral tissue depths confirmed a thermal gradient from the hotter core to the cooler periphery (Whitby and Dunkin, 1971; Mellergard and Nordstrom, 1990; Hirashima et al., 1998).

CSF acts as a CNS temperature buffer and may have more extensive thermal interactions with the superficial cerebral tissues (<2–3 cm) via the strikingly vast CSF-cortical and CSF-vessel contact surface areas (see below). In addition, it is thought that the superficial cerebral tissues may be more susceptible thermally to the external environment. In humans, brain temperatures measured in the superficial cerebral tissues, including the subdural and epidural spaces, can be lower than body temperatures (Mellergard and Nordstrom, 1990; Hirashima et al., 1998; Nakagawa et al., 2011; Suehiro et al., 2011).

Intraoperative infrared imaging of surgically-exposed human cortex demonstrated baseline cortical temperature heterogeneity as well as reproducible temperature increases within the primary somatosensory cortex during median nerve stimulation, and the sensorimotor cortex during repetitive hand movements and finger tapping, and also in language areas during speech production (Gorbach et al., 2003). The detected temperature increases (0.04–0.08°C) with various stimuli were quite small compared with animal data obtained using thermoresistors (thermistors) or thermocouples implanted into the brain (Serota, 1938, 1939a; Hamilton, 1963; Hammel et al., 1963; Jackson and Hammel, 1963; Kawamura and Sawyer, 1965a; Hemingway et al., 1966; Delgado and Hanai, 1966b; Hayward and Baker, 1968b; Reite and Pegram, 1968; Kovalzon, 1973; Kiyatkin et al., 2002), but were similar to rodent data acquired with infrared imaging (Shevelev, 1998). Implanted thermoresistors (relying on temperature dependent changes in resistance between two leads) and thermocouples (relying on the Seebeck effect) may thus exhibit a quantitative but not a qualitative difference vs. less invasive techniques based on imaging.

Ecker et al. (2002) used infrared cameras to detect temperature changes of the cerebral cortex during awake craniotomy in humans (Figure 2). The authors reported correlations between the temperature of cortex with various stimuli, such as speech cortex with verbalization, and motor cortex with hand motion. A 0.7°C temperature increase of the speech cortex during verbalization was reported.

Magnetic resonance imaging (MRI) has evolved as a valid noninvasive technique to characterize brain temperature. With functional magnetic resonance imaging (fMRI), decreases in the local brain temperature on the order of 0.2°C were reported in the visual cortex during prolonged visual stimulation of human brain (Yablonskiy et al., 2000). The study results contradict the majority of animal and human data concerning regional brain temperature changes during functional activation. fMRI relies on the changes in the signal intensity due to blood oxygen level-dependent (BOLD) contrast. The BOLD effect depends upon a complex interaction between temperature dependent variables, such as the oxidation of blood hemoglobin, blood flow, and cerebral metabolism (Ogawa et al., 1990, 1993). Therefore, the discrepancy may have stemmed from the current methodological limitations in measuring these temperature-dependent variables and makes it difficult to determine whether fMRI has accuracy to match its precision.

## Brain temperature and parenchyma

### Cerebral tissue sensitivity and tolerance to temperature changes

CNS tissue is one of the most sensitive to heat (Haveman et al., 2005). Both the change in temperature (ΔT) and the duration of exposure are critical in determining the degree of thermal tolerance as well as the amount of thermal damage to the tissue. Although studies using chronic implants of a cooling loop demonstrated that repeated cooling did not alter neuronal responsiveness, animal behavior, or produce anatomical damage (Keating and Gooley, 1988; Lomber et al., 1996, 1999; Fingas et al., 2007), very little is known about human cerebral tissue's response and tolerance to chronic exposure to a thermal stress (e.g., cortical brain-machine interface or implanted cooling patch) that generates low (<2°C) temperature changes.

Permeability of the blood-brain barrier (BBB), quantitatively evaluated via intra-brain albumin leakage, is highly temperature dependent (Kiyatkin and Sharma, 2009). In rats, the number of albumin-positive cells were noted to be minimal at normothermic values (34.2–38°C), slightly higher (2–4 fold) at hypothermic values (32.2–34.2°C), and dramatically higher (26 fold) at hyperthermic values (38–42.5°C). Brain cells are exceptionally sensitive to thermal damage. In cultured neurons, spontaneous activity stopped irreversibly between 42 and 43°C (Gahwiler et al., 1972) indicating a direct cell-level injury. In a rat model, structurally abnormal cells were absent at low and normal temperatures (Kiyatkin and Sharma, 2009). Although a few abnormal cells were found at 38.5°C, the number increased linearly as temperature rose (Kiyatkin and Sharma, 2009). The temperature threshold to produce progressive thermal injury to the metabolically active brain cells, BBB, and the vascular endothelium appears to be between 39 and 40°C (Bechtold and Brown, 2003; Sharma and Hoopes, 2003; Kiyatkin and Sharma, 2009). At a similar temperature threshold, heat-shock proteins are activated to induce heat tolerance and enhance cellular protection (Haque et al., 2012). In various settings of physiological hyperthermia (38.5–39.5°C) such as environmental warming, intense physical exercise, and copulatory behavior, an increase in BBB permeability is well-noted, and emerging research indicates that hyperthermia within the physiological range may potentiate cerebral damage induced by pathological processes such as trauma and stroke (Hajat et al., 2000; Jiang et al., 2002; Dietrich and Bramlett, 2010).

In addition to damage at the cellular level, thermal damage is also a part of tissue-level sequelae that includes parenchymal edema and damage to the BBB. Chronic ischemic conditions are known to damage not only the vascular but also the ventricular side of the BBB, with autophagy in endothelial cells and pericytes alike and astrogliosis in nearby parenchyma (Garbuzova-Davis et al., 2014), and it has been postulated that enhancement of venous drainage from an ischemic site could improve patient outcome (Li et al., 2014). New evidence suggests that well-known blood pressure lowering drugs, specifically angiotensin II type two receptor blockers, can preventatively block edema and subsequent parenchymal damage to the brain by blocking the damage to the BBB (Min et al., 2014). While these studies deal with injury secondary to ischemia, the pathophysiological effects of dysregulation of oxidative phosphorylation are similar whether the inciting event is a an oxygen supply decrease or a more direct dysregulation of mitochondrial proteins such as UCP-2 and generally result in the same increased ROS and mitochondrial membrane permeability. Cardiac researchers have recognized this and made gains either through utilization of hypothermia (Zhou et al., 2011) or drugs that reduce ROS generation more specifically (Deja et al., 2009). The use of drugs with selective physiological effects is an approach not lost on neuroscienctists (Gheibi et al., 2014), nor is selectively applied hypothermia (Wang et al., 2004).

The cerebral tissue appears to have a remarkable tolerance to brain temperature reduction. In patients undergoing operative deep hypothermic circulatory arrest (Mascio et al., 2009; Percy et al., 2009), the brain appears to tolerate temperatures of 15–18°C well for relatively short intervals (2–3 h). Yang et al. (2003) cooled cat brains to 3°C for 1–2 h daily for 7–10 months and demonstrated no significant neuropathological consequences. Cooling rat brains over the sensorimotor cortex, Oku et al. (2009) demonstrated that focal brain temperature reduction above 0°C for 1 h did not result in motor dysfunction or histological changes. However, cooling at −5°C for 1 h resulted in transient motor dysfunction and irreversible histological changes (Oku et al., 2009). Using electroencephalography (EEG) (Schiller et al., 1974) or evoked potentials elicited by sensory (Jasper et al., 1970; Kalil and Chase, 1970) or electrical stimulation (Benita and Conde, 1972; Schmielau and Singer, 1977), studies on neural inactivation by the cold demonstrated that the EEG and evoked potentials did not disappear until around 10°C. In the Macaque monkey, the cortical neurons did not cease to respond to visual stimulation until they were cooled to temperatures between 4 and 18°C (Girard and Bullier, 1989). *In vitro* studies using guinea pig hippocampal slices demonstrated that synaptic function, neuronal excitability, and membrane properties, although greatly influenced by local temperature reduction, maintained their reversibility after > 90 min of cooling at temperatures < 10°C (Aihara et al., 2001). Gahwiler et al. (1972) demonstrated in cultured neurons that all neurons were still active at 20°C and that spontaneous activity remained in a number of neurons for temperatures between 5 and 10°C.

### Implications for molecular physiology of the synapse

Brain temperature may be an important but non-specific and passive physiological parameter with its fluctuations determined primarily by alterations in neuronal activity, brain metabolism, and CBF. Recent evidence, however, indicates that small temperature gradients generated in axon terminals upon the activation of brain uncoupling proteins (UCPs) may directly modulate presynaptic and postsynaptic events, as neurotransmitter diffusion and convection are temperature dependent (Horvath et al., 1999; Andrews et al., 2005; Fuxe et al., 2005). The UCPs, as a family of mitochondrial anion-carrier proteins, are located in the inner membrane of mitochondria. Activation of UCPs reduces the mitochondrial membrane potential by causing a proton leak back into the matrix. This process dissipates the proton motive force, uncouples oxidative phosphorylation from ATP synthesis, and thereby generates heat and local temperature gradients. UPCs are relatively well understood in the context of brown adipose tissue, specifically UPC-1, in which they generate heat associated with vasodilation (Matthias et al., 2000) and increases in glucose consumption in excess of that calculated from increased blood flow alone (Wang et al., 2006b), indicative of active uptake and utilization of glucose for heat production confirmed through nuclear medicine studies (Cypess et al., 2013). In the case of the brain, this distinction between passive warming through increased blood flow and active warming through energy utilization is more difficult to elucidate but recent evidence suggests this may be accomplished by UPCs. This highly provocative hypothesis that CNS synaptic transmission may be modulated by heat produced locally in axon terminals still warrants experimental investigation.

What is known is that a UPC variant, UPC-2, is expressed in a unique manner in neural tissue (Richard et al., 1998; Horvath et al., 1999) despite an overall wide distribution (Fleury et al., 1997). Immunohistochemical staining of human tissue preparations demonstrate the localization of UPC-2 predominantly to axons and axon terminals (Horvath et al., 1999). Thus, the ability to generate heat in neurons is concentrated in axons and axon terminals, indicating that heat generation may be intricately involved in neurotransmission either as a modulatory factor or as a direct response to a stimulus. As a sequence of coupled diffusive and reactive events, synaptic transmission involves molecular processes that display stochastic (random) properties (Ribrault et al., 2011). At the synapse, Brownian motion governs not only the diffusion of small molecules such as neurotransmitters and calcium ions, but also the motion of proteins such as receptors or scaffolding proteins within the membrane or the cytoplasm (Triller and Choquet, 2008). Brownian motion, as the intrinsic random motion of molecules, is characterized by a diffusion coefficient that is fundamentally temperature dependent (Ribrault et al., 2011).

Although findings from adipose tissue indicate that regional temperature differences are not attributable to bulk flow variance but rather to intrinsic heat-generating activity, it cannot be assumed that temperature variance in neural tissue is directly related to—let alone scales with—neural activity levels. While temperature variance may indeed be an important variable influencing neural transmission, more experimentation is needed to determine the functional importance of temperature at the synapse.

Recent evidence suggests that neural transmission may be a function of thermodynamic energy barriers to physiological mechanisms (Rost et al., 2011) which are as yet not understood. For example, one such physiological barrier is the well-known case of vesicular fusion at axon terminals, the biophysical rate-limiting step of neurological transmission. Of all the barriers to efficient synaptic transmission, vesicular fusion stands out for its biophysical rather than statistical nature. The process of union of two phospholipid bilayers has a high entropic barrier, and is dependent upon SNARE proteins to overcome the energy barrier of activation. Owing largely to their amphipathic nature, the discovery of the SNAREs helped to reveal how the major thermodynamic barrier to neurotransmitter release might be overcome through enzymatic intermediate steps, and to explain how such entropically counter-intuitive steps could happen very rapidly and repeatedly (Trimble et al., 1988).

As the energy derived from SNARE proteins is dependent only upon Boltzmann's constant and temperature (Gao et al., 2012), increased temperatures at axon terminals are strongly implicated as a modulatory factor capable of regulating the process of vesicle fusion through modulation of whether this energetic barrier can be overcome. Indeed, in well-studied multiple-input systems such as hippocampal neurons, metabotropic GABA-B receptors have been shown to inhibit neurotransmitter release by increasing the energy barrier to synaptic transmission (Rost et al., 2011), a gap which recent SNARE research suggests could be overcome with increased local temperature (Gao et al., 2012). The fact that this phenomenon has been shown to happen in hippocampal neurons is suggestive of its involvement in complex neural circuits, perhaps indicating that axonal heat variance is a technique employed by highly active centers with multiple integrating influences to globally modulate signal transmission in axons analogous to long-term potentiation and depression (LTP/LTD). Due to the passive radiation of heat, such a temperature-based phenomenon would be potentially applicable both to single synapses and to local regions over which locally generated heat could dissipate. It is possible that temperature variation may be employed to further fine-tune activity-dependent processes such as learning and memory, as exemplified by the hippocampus.

## Brain temperature and cerebral circulation

### Cerebral circulation and the thermal environment of the brain

The net chemical reaction of oxygen and glucose generates most of the energy required for cerebral metabolic activities. While some of this energy (33%) is immediately released as heat, the rest is used to produce ATP molecules to fuel a complex chain of chemical reactions (Siesjö, 1978). Given that no mechanical work is performed in this process, the final ATP hydrolysis releases the energy back to the biological system as heat (Siesjö, 1978). On average, 0.66 J is released every minute per gram of brain tissue (Yablonskiy et al., 2000). If not promptly removed, this heat generation and accumulation will lead to a continuous increase in local brain temperature. In humans and other large animals, the principal heat removal mechanism is through the cerebral circulation.

Cerebral circulation is most critical for stabilizing the thermal environment of the brain. With compromised cerebral circulation, the heat exchange properties of the brain rapidly change. In a monkey model of cardiac arrest occurring at 35°C ambient temperature, superficial brain sites immediately cooled while deep brain temperatures initially rose. However, after 8–10 min, the deeper intracranial sites cooled in parallel to surface temperatures (Hayward and Baker, 1969). Increasingly ambient air temperature to 45°C eliminated this pattern of immediate cooling of frontal subcortical sites and later cooling of all brain sites (Hayward and Baker, 1969).

In awake or anesthetized large animals, the physiological direction of thermal energy flow is from the brain (heat source) to the blood (heat sink). Cooling or warming of the perfusing blood results in prompt thermal exchange with various brain sites. In regards to speed and amount of heat transfer, it is most effective in the cerebral cortex adjacent to a cortical arteriole (Hayward and Baker, 1968b). With no significant change in metabolic heat production, vasodilatation enhances cerebral heat clearance, while vasoconstriction impedes brain cooling (Hayward and Baker, 1969). Known to produce cerebral vasodilatation, hypercapnia, induced in monkeys using 8–10% CO_2_ in inhalation air, narrows the brain-body temperature gradients. Similarly, hypocapnia, induced with hyperventilation, widens the brain-body temperature gradients. In anesthetized small animals such as rats, although the brain-body temperature gradient is negative with the absolute value being dependent on experimental circumstances (see above), hypercapnia-induced cerebral vasodilatation elevates the absolute brain temperature and subsequently narrows the brain-body temperature gradient (Zhu et al., 2009).

In humans, cortical arterial branches traverse over long distance (up to 20 cm) within the subarachnoid space. They are thin-walled vessels with diameters similar to those of the carotid rete of other species (Simoens et al., 1987). Because of the structural similarities to the carotid rete, cortical arterial vessels are thought to have highly effective CSF-arterial thermal interaction (Zenker and Kubik, 1996). In addition, 70–80% of the cerebral blood volume circulates in the veins. Like the cortical arterial vessels, cortical veins are also thin-walled and traverse over long distance within the subarachnoid space. In contrast to cortical arterial vessels, the cortical veins contain cerebral blood circulating at low velocity and pressure. The venous vasculature, like the arterial vasculature, likely provides a highly effective CSF-venous thermal exchange interface and regulation checkpoint.

### Brain temperature and cerebral blood

Brain temperature and CBF are two closely associated homeostatic parameters with significant mutual impact. CBF is the principal mechanism to maintain brain and body temperature coupling; physiological or pathological perturbations of CBF may result in significant changes of brain temperature with the subsequent de-coupling of brain and body temperature (Hayward and Baker, 1968b; Zhu et al., 2009). Yet, the interplay of regional CBF and local brain temperature regulation has received scant attention.

The pattern of brain temperature changes (global vs. regional), the magnitude of brain temperature changes (physiological vs. pathological), and the underlying induction mechanism (extravascular vs. intravascular) must be considered in delineating the corresponding effects on CBF. An induction mechanism that relies primarily on convective heat exchange through the cerebral circulation to accomplish brain temperature alterations is considered intravascular in nature. For example, whole body hypo- or hyper-thermia secondarily induces brain temperature changes convectively through the cerebral circulation and is therefore intravascular in nature. Temperature manipulation of the perfusing blood in the cervical carotid arteries to accomplish selective brain cooling or warming is also intravascular in nature. Conversely, extravascular induction mechanisms do not primarily rely on convective heat exchange through the cerebral circulation. They include conductive (e.g., heating or cooling devices applied externally), convective (e.g., forced cold or hot air circulation), and other methods (e.g., high intensity exposure to radiofrequency electromagnetic fields).

Brain hypothermia secondarily induced through whole body cooling has been associated with a reduction in CBF (Hagerdal et al., 1975; Busija and Leffler, 1987; Greeley et al., 1991; Michenfelder and Milde, 1991; Laptook et al., 2001). Significant brain temperature elevation associated with whole body hyperthermia during intense exercise is well documented in humans (Saltin et al., 1972; Nybo et al., 2002b), and this thermal perturbation also results in a reduction of CBF (Nybo and Nielsen, 2001; Nybo et al., 2002a,b). This reduction is only partly related to the hyperventilation induced decline in arterial P_CO2_. Due to the confounding effects of systemic hypothermia or hyperthermia on the cardiovascular and respiratory systems, it is difficult to elucidate the relationship between brain tissue temperature and CBF using whole body temperature manipulation methodologies. Therefore, selective brain cooling or warming may be a more suitable experimental condition to further delineate the regulatory mechanisms between brain tissue temperature and CBF.

The limited numbers of studies investigating the cerebral hemodynamic consequences of selective brain cooling have yielded contradictory results. Walter et al. (2000) induced selective brain cooling in juvenile pigs by bi-carotid perfusion of the head with extracorporally cooled blood and demonstrated a reduction of CBF to a degree comparable to whole body cooling. Similar findings were reported in baboons with extracorporally cooled blood through one carotid artery (Schwartz et al., 1996). Gelman et al. (1996) demonstrated no change in CBF at 15 and 45 min of selective brain cooling after cardiac arrest in infant piglets. In newborn miniature swine, selective brain cooling resulted in a reduction of global CBF (Laptook et al., 2001). In rats, Kuluz et al. (1993) used laser-Doppler flowmetry (LDF) and reported a significant increase in cortical CBF (to 215% of baseline values) during selective brain cooling. The conflicting findings of these studies suggest that cerebrovascular response to brain cooling may depend on differences in species (particularly rete vs. non-rete species), age, and induction mechanisms (e.g., intravascular vs. extravascular and global vs. regional).

The relevance of regional CBF and local brain temperature is well recognized (Moriyama, 1990; Kuluz et al., 1993; Ohmoto et al., 1996; Masuda et al., 2011). In the skin, temperature changes result in local skin blood flow changes through autoregulated microcirculation (Hales et al., 1978; Charkoudian, 2003). Similar microcirculation has also been identified in the human brain cortex (Duvernoy et al., 1981). Therefore, cortical temperature changes may regulate regional CBF through autoregulation of the microcirculation. Temperature induced alterations in local cerebrovascular resistance has also been proposed as a possible mechanism for the corresponding changes in regional CBF (Kuluz et al., 1993). Moriyama (1990) demonstrated that regional CBF changes depended upon the cortical temperature alterations in the 36–46°C range in monkeys. In rats, Masuda et al. (2011) induced cortical temperature changes with radiofrequency electromagnetic fields and demonstrated temperature-dependent regulation of regional CBF in the 33–50°C range. Under physiological conditions, in the absence of cell damage, regional CBF elevation correlates with cortical temperature rise in a linear fashion (Masuda et al., 2011). However, prolonged temperature elevation (≥20 min) above 43°C resulted in the gradual decline in CBF (Moriyama, 1990; Ohmoto et al., 1996) and whether such a linear relationship exists with physiologic changes in humans remains in question.

In human studies changes in regional CBF have been linked to states of maladaptive responses to normal stimuli (Bradley et al., 2000), a finding confirmed by different imaging techniques (Sundgren et al., 2007; Duschek et al., 2012) but whose clinical applicability is currently debated (Guedj, 2009). Caution must be used when evaluating these studies, however, as some (Inamura et al., 2013) use sub-physiological temperatures known to trigger the disassembly of microtubules thereby potentially undermining endothelial cell structure and disrupting basic cellular functions. Results would then be subject to uncertainty with respect to what cellular responses are specific to the process being investigated. Most studies recognize the dangers of studying physiological processes at such temperatures and generally accept temperatures below 29°C as “profound hypothermia” (Dietrich et al., 2009). In the case of inflammatory processes, cytokine-mediated increased vascular permeability results in higher blood flow to the area of inflammation and classically contributes to local temperature increases, known to Celsius as *calor*. It does not necessarily follow, however, that increased local temperature is a function of increased blood flow.

It is more likely that CBF and brain temperature relate to each other in a complicated fashion. To date, the regulatory mechanism of CBF, as one of the central pieces of the neurophysiology puzzle, remains elusive. Supportive evidence for a number of established hypotheses is based on *in vitro* experiments involving isolated tissue: brain slices, excised vessels, cell cultures, etc. (Raichle and Mintun, 2006; Attwell et al., 2010; Cauli and Hamel, 2010; Paulson et al., 2010). Although *in vitro* experiments have produced a wealth of data, extrapolation of conclusions to the *in vivo* situation remains uncertain. Many of the *in vivo* physiological parameters and intracellular milieu cannot be realistically simulated *in vitro*. In particular, CBF and brain tissue temperature represent two of such essential parameters that depart significantly between the *in vitro* and *in vivo* conditions. For example, experiments using brain slices cannot examine CBF and are typically conducted at significantly sub-physiological temperatures due to the tissue vitality concerns. With the advent of new technologies to image brain temperature, CBF, and metabolism, efforts directed toward establishing an *in vivo* model are crucial to further elucidate the regulatory mechanisms between CBF and brain temperature.

### Brain temperature regulation and stress states

Neuronal stress is not a superficial or transient change in cellular function, but a fundamental and multifaceted alteration of cellular physiology (Khatri and Man, 2013) which affects intracellular homeostatic physiology and intercellular system physiology (Bartnik-Olson et al., 2013). What is especially provocative about states of neuronal stress, under certain circumstances culminating in neurodegenerative disease, is the recurrence of amphipathic proteins recognized as causal agents in several such diseases. Amphipathic proteins are known to have activities highly sensitive to temperature due ti its influence on hydrophobic/hydrophilic interactions. The amphipathicity of these proteins suggests an intrinsic responsiveness to temperature, but the physiological significance of neural temperature regulation or impact upon specific proteins remains unclear. Further, there is evidence that the modulation of biophysical parameters is an underappreciated method of neurotransmitter regulation. SNARE proteins are of such importance in large part because of their amphipathic nature. Several other examples of neuronal amphipathic proteins are known, including alpha-synuclein which displays increased expression in neurons involved in learning pathways in songbird systems (George et al., 1995) and experience-dependent turnover of synaptic vesicles within axons (Clayton and George, 1999), suggesting history-dependent modulation similar to that observed in LTP/LTD. Alpha-synuclein is better known for its role in pathologies, however, most notably Parkinson Disease among a host of so-called synucleinopathies known to involve alpha-synuclein dysfunction though precise mechanisms have yet to be elucidated (Cheng et al., 2011). Another amphipathic protein of note shares several features with alpha-synuclein, most notably secondary structure (Serpell et al., 2000) and an involvement in both physiology and pathophysiology: amyloid beta. Known best for its characteristic involvement in Alzheimer Disease, amyloid beta also is involved in acute responses to trauma as shown in murine models (Marklund et al., 2013) and cellular stress in general as shown clinically (Abrahamson et al., 2006). In human specimens, amyloid beta and amyloid precursor protein (APP) accumulate after traumatic brain injury despite the lack of any appreciable plaques in long-term studies (up to 3 years) (Chen et al., 2009). A similar study found that in human deaths from traumatic brain injuries, not only amyloid beta and APP but also alpha synuclein accumulated in neural tissue (Chen et al., 2004), lending credence to the prevailing theory that neurodegenerative disease represents an inappropriate activity of a physiological mechanism triggered by stress. The recurrence of amphipathic proteins in such pathologies is suggestive of a role for temperature in neural regulatory patterns, and recent research into neural UPCs lends some of the most specific such evidence.

Increased levels of UPC-2 have been found in neurons experiencing physiological stress such as chronic alcohol consumption, in which the compensatory increase of UPC-2 initially reduces neuronal cell death but eventually reaches toxic levels due to its involvement in the mitochondrial permeability transition pore (mPTP) (Graw et al., 2013). The mPTP complex causes cell death primarily through interference of complex I of the electron transport chain in mitochondria. Malfunctions of other specific components of the mPTP have also been linked to disease states such as myositis, Paget disease and frontotemporal dementia in addition to approximately 1–2% of all cases of amyotrophic lateral sclerosis (ALS) (Bartolome et al., 2013). Mitochondrial uncoupling is known to reduce ATP levels and increase reactive oxygen species; further, mitochondrial depolarization and dysfunction has been shown to be present in a host of neurodegenerative diseases (Chaturvedi and Beal, 2013; Chaturvedi and Flint Beal, 2013) and is thought to be a contributor to the etiology of such diseases. A particularly interesting correlation is seen with alpha-synuclein, which has been shown to interact with the mPTP in a fashion similar to UPC-2, specifically interacting with a voltage-dependent pore component VDAC1 and facilitating a transition to apoptosis at high activity levels (Lu et al., 2013). This pattern of an initially compensatory reaction to a stimulus turning to a detrimental reaction once sustained is curiously common, not only with respect to neurodegenerative disease hypotheses and chronic alcohol consumption discussed above but also to other systemic physiological disease states.

The field of cardiology has been dealing with the issue of ischemia-reperfusion injury for some time (Robinson et al., 2011). Interest in mitochondrial uncoupling proteins specifically (Murray et al., 2004) and cellular damage through ROS and mitochondrial dysregulation in general (Yusuf et al., 2012) has lead to some interesting discoveries in pathogenesis and possible pharmacological therapies (Deja et al., 2009; Wang et al., 2010). It has long been recognized (Mattson and Kroemer, 2003) that some of the same basic dysfunctions underlie the pathogenesis of both cardiac and neural end organ damage: mitochondrial homeostasis (Rajanikant et al., 2007; Sharma et al., 2013), ROS generation (Wang et al., 2006a; El-Sawalhi and Ahmed, 2014), and inadequate ROS scavenging (Green and Ashwood, 2005; Sabbah et al., 2013). The pharmalogical benefits of any number of antioxidant moieties have been studied and some have shown promise for cardiac and neural dysfunction alike (Green and Ashwood, 2005; Mack et al., 2006; Wang et al., 2006a; Hernandez-Resendiz et al., 2013) in a rapidly expanding field of literature. A full review of the pharmacology is regrettably not within the scope of the present article.

## Anatomical consideration

### Surrounding structures

In order to effectively study and examine brain temperature regulation, it is critical to understand the relevant anatomical and physiological properties in the head-neck regions. Heat transfer in the head occurs via radiation, conduction, convection, and evaporation and the various anatomical features have different thermal properties.

The bony and soft coverings of the brain are composed of head hair, scalp, skull, and meninges. Collectively, those structures help maintain brain temperature homeostasis by shielding the brain from external thermal challenges. Without these layers, the surgically exposed cerebral cortex can have temperatures 5–10°C below core body temperatures (Gorbach et al., 2003; Kalmbach and Waters, 2012). The surgical removal of a large piece of skull, performed to relieve the intracranial pressure after brain injury, increased brain's thermal susceptibility to the external environment and resulted in lowered brain temperatures (Nakagawa et al., 2011; Suehiro et al., 2011). An additional structural feature of the head is its round shape, which minimizes surface area to volume ratio and therefore further protects the brain from environmental thermal challenges.

In humans diagnosed with brain death, despite normal body core temperature, the scalp cools significantly because of the lack of metabolic thermal load generated by the cerebrum. In children, a rectal-scalp temperature difference of greater than 4°C has been reported to correlate with clinical criteria for brain death (Miller et al., 1999). In primates, although temperatures of deep brain sites as well as the arterial blood were unaffected by environmental cooling or warming, shifts in air temperature from 5 to 7°C on either side of the neutral zone (28- and 32°C) were reported to increase or decrease temperatures of superficial cerebral sites and CSF of the basal subarachnoid space (Hayward and Baker, 1968b). These data illustrate that heat exchange with the environment through the overlying skull and scalp is a compensatory physiological mechanism of thermal transfer by the brain, particularly for the cortical surface. However, under physiological conditions with normal anatomical arrangements, the ambient temperature is not likely to thermally impact the cerebral tissue beyond the depth of 2–3 cm from the cortical surface (Stone et al., 1997; King et al., 2010) without temperature changes in the perfusing blood.

### Cerebrospinal fluid

The CSF provides a second fluid circulation system for the CNS, analogous to the lymphatic system for the rest of the body (Cushing, 1914; Taketomo and Saito, 1965; Milhorat, 1975). The fluid around the brain provides a suspensive force, reducing the brain's effective weight from 1500 g to only 50 g (Segal, 1993). CSF is vital to the brain's structural, biochemical, and thermal health (Wolfson et al., 1974; Segal, 1993; Zenker and Kubik, 1996; Redzic et al., 2005; Johanson et al., 2008).

CSF fills the brain ventricles internally, and the subarachnoid space externally. It intimately interacts with the blood circulation not only in the subarachnoid space but also at the capillary level through the perivascular spaces (formally called Virchow-Robin spaces) and the interstitial spaces (Hutchings and Weller, 1986; Esiri and Gay, 1990; Zhang et al., 1990). The contact surface between the CSF and the brain is extraordinarily large. For example, in adult humans, the cerebrocortical surface area was approximated to be 2300 cm^2^ (Elias and Schwartz, 1969). Inside the brain, the fluid-brain contact surface at the capillary level through the perivascular spaces and the interstitial spaces is even more extraordinary, estimated at 250 cm^2^/g of the tissue (Crone, 1963; Raichle, 1983). The strikingly vast fluid-brain and fluid-vessel contact surface is essential for CSF to stabilize and finely regulate the biochemical and thermal environment of the CNS.

Multiple studies using CSF tracers have confirmed that the fluid spaces of the subarachnoid, ventricular, perivascular, and interstitial compartments constitute a functional continuity (Wagner et al., 1974; Rennels et al., 1990; Stoodley et al., 1997; Brodbelt et al., 2003); therefore, fluid-brain and fluid-vessel thermal interaction at the capillary level must also be inferred.

## Conclusion

A synthesis of brain temperature regulation and the effects of brain hypo- and hyper-thermia is needed not just for a better understanding of neurophysiology, but also to achieve better outcomes for patients with common neurological or cardiac injuries/illnesses. For example, the current standard of care in patients at risk for myocardial infarctions is focused on reducing infarct size, but tissue damage resulting from reperfusion injury may be limiting the utility of such proven beneficial measures as cardiopulmonary resuscitation (CPR) (Neumar et al., 2008). The research reviewed in this article illustrates the potential for improved outcomes, and as Nielsen et al. (2013) recently demonstrated the lack of harm from pre-hospital cooling, the field may now shift focus from determining whether cooling might improve patient outcomes to how best to implement and standardize cooling procedures to minimize post-CPR reperfusion injury on return of spontaneous circulation (Rittenberger and Callaway, 2013). Some problems associated with early clinical studies of pre-hospital cooling appear method dependent, such as transient pulmonary edema in patients cooled with a 2L 4°C saline infusion (Kim et al., 2013), again indicating that optimization of currently used methods or experimentation into new methods of cooling would be beneficial. There have already been calls to open up such optimization with more and larger trials, especially with respect to better defining neurological outcomes in myocardial infarct cases by instituting a protocol for withdrawal of care (Rittenberger and Callaway, 2013).

Humans, as endotherms, maintain a nearly constant core-body temperature (36–37.5°C) over a wide range of environmental conditions. For the past two decades, CNS temperature, as an independent therapeutic target variable, has received increasingly intense clinical attention. Above, we have discussed several critical aspects concerning the fundamental properties of brain temperature. We will continue to direct our subsequent efforts to best summarize the current state of knowledge on CNS temperature from a clinical perspective.

### Conflict of interest statement

The authors declare that the research was conducted in the absence of any commercial or financial relationships that could be construed as a potential conflict of interest.

## References

[B1] Abbott B. C. Hill A. V. Howarth J. V. (1958). The positive and negative heat production associated with a nerve impulse. Proc. R. Soc. Lond. B Biol. Sci. 148, 149–187 10.1098/rspb.1958.0012 13518134

[B2] Abrahamson E. E. Ikonomovic M. D. Ciallella J. R. Hope C. E. Paljug W. R. Isanski B. A. (2006). Caspase inhibition therapy abolishes brain trauma-induced increases in Abeta peptide: implications for clinical outcome. Exp. Neurol. 197, 437–450 10.1016/j.expneurol.2005.10.011 16300758

[B3] Aihara H. Okada Y. Tamaki N. (2001). The effects of cooling and rewarming on the neuronal activity of pyramidal neurons in guinea pig hippocampal slices. Brain Res. 893, 36–45 10.1016/S0006-8993(00)03285-6 11222990

[B4] Andalman A. S. Foerster J. N. Fee M. S. (2011). Control of vocal and respiratory patterns in birdsong: dissection of forebrain and brainstem mechanisms using temperature. PLoS ONE 6:e25461 10.1371/journal.pone.0025461 21980466 PMC3182229

[B5] Andersen P. Moser E. I. (1995). Brain temperature and hippocampal function. Hippocampus 5, 491–498 10.1002/hipo.450050602 8646277

[B6] Andrews Z. B. Diano S. Horvath T. L. (2005). Mitochondrial uncoupling proteins in the CNS: in support of function and survival. Nat. Rev. Neurosci. 6, 829–840 10.1038/nrn1767 16224498

[B7] Aronov D. Fee M. S. (2012). Natural changes in brain temperature underlie variations in song tempo during a mating behavior. PLoS ONE 7:e47856 10.1371/journal.pone.0047856 23112858 PMC3480430

[B8] Attwell D. Buchan A. M. Charpak S. Lauritzen M. Macvicar B. A. Newman E. A. (2010). Glial and neuronal control of brain blood flow. Nature 468, 232–243 10.1038/nature09613 21068832 PMC3206737

[B9] Bartnik-Olson B. L. Harris N. G. Shijo K. Sutton R. L. (2013). Insights into the metabolic response to traumatic brain injury as revealed by C NMR spectroscopy. Front. Neuroenergetics 5:8 10.3389/fnene.2013.00008 24109452 PMC3790078

[B10] Bartolome F. Wu H. C. Burchell V. S. Preza E. Wray S. Mahoney C. J. (2013). Pathogenic VCP mutations induce mitochondrial uncoupling and reduced ATP levels. Neuron 78, 57–64 10.1016/j.neuron.2013.02.028 23498975 PMC3843114

[B11] Bechtold D. A. Brown I. R. (2003). Induction of Hsp27 and Hsp32 stress proteins and vimentin in glial cells of the rat hippocampus following hyperthermia. Neurochem. Res. 28, 1163–1173 10.1023/A:1024268126310 12834255

[B12] Benita M. Conde H. (1972). Effects of local cooling upon conduction and synaptic transmission. Brain Res. 36, 133–151 10.1016/0006-8993(72)90771-8 4332736

[B13] Bernard S. A. Gray T. W. Buist M. D. Jones B. M. Silvester W. Gutteridge G. (2002). Treatment of comatose survivors of out-of-hospital cardiac arrest with induced hypothermia. N. Engl. J. Med. 346, 557–563 10.1056/NEJMoa003289 11856794

[B14] Bradley L. A. Mckendree-Smith N. L. Alberts K. R. Alarcon G. S. Mountz J. M. Deutsch G. (2000). Use of neuroimaging to understand abnormal pain sensitivity in fibromyalgia. Curr. Rheumatol. Rep. 2, 141–148 10.1007/s11926-000-0054-2 11123051

[B15] Brodbelt A. R. Stoodley M. A. Watling A. M. Tu J. Jones N. R. (2003). Fluid flow in an animal model of post-traumatic syringomyelia. Eur. Spine J. 12, 300–306 10.1007/s00586-002-0492-9 12800004 PMC3615493

[B16] Brooks V. B. (1983). Study of Brain Function by Local, Reversible Cooling Reviews of Physiology, Biochemistry and Pharmacology, Vol. 95 Berlin; Heidelberg: Springer, 1–109

[B17] Busija D. W. Leffler C. W. (1987). Hypothermia reduces cerebral metabolic rate and cerebral blood flow in newborn pigs. Am. J. Physiol. 253, H869–H873 3661735 10.1152/ajpheart.1987.253.4.H869

[B18] Cauli B. Hamel E. (2010). Revisiting the role of neurons in neurovascular coupling. Front. Neuroenergetics 2:9 10.3389/fnene.2010.00009 20616884 PMC2899521

[B19] Charkoudian N. (2003). Skin blood flow in adult human thermoregulation: how it works, when it does not, and why. Mayo Clin. Proc. 78, 603–612 10.4065/78.5.603 12744548

[B20] Chaturvedi R. K. Beal M. F. (2013). Mitochondria targeted therapeutic approaches in Parkinson's and Huntington's diseases. Mol. Cell. Neurosci. 55, 101–114 10.1016/j.mcn.2012.11.011 23220289

[B21] Chaturvedi R. K. Flint Beal M. (2013). Mitochondrial diseases of the brain. Free Radic. Biol. Med. 63, 1–29 10.1016/j.freeradbiomed.2013.03.018 23567191

[B22] Chen X. H. Johnson V. E. Uryu K. Trojanowski J. Q. Smith D. H. (2009). A lack of amyloid beta plaques despite persistent accumulation of amyloid beta in axons of long-term survivors of traumatic brain injury. Brain Pathol. 19, 214–223 10.1111/j.1750-3639.2008.00176.x 18492093 PMC3014260

[B23] Chen X. H. Siman R. Iwata A. Meaney D. F. Trojanowski J. Q. Smith D. H. (2004). Long-term accumulation of amyloid-beta, beta-secretase, presenilin-1, and caspase-3 in damaged axons following brain trauma. Am. J. Pathol. 165, 357–371 10.1016/S0002-9440(10)63303-2 15277212 PMC1618579

[B24] Cheng F. Vivacqua G. Yu S. (2011). The role of alpha-synuclein in neurotransmission and synaptic plasticity. J. Chem. Neuroanat. 42, 242–248 10.1016/j.jchemneu.2010.12.001 21167933

[B25] Clayton D. F. George J. M. (1999). Synucleins in synaptic plasticity and neurodegenerative disorders. J. Neurosci. Res. 58, 120–129 10491577

[B26] Clifton G. L. Miller E. R. Choi S. C. Levin H. S. Mccauley S. Smith K. R. Jr. (2001). Lack of effect of induction of hypothermia after acute brain injury. N. Engl. J. Med. 344, 556–563 10.1056/NEJM200102223440803 11207351

[B27] Clifton G. L. Valadka A. Zygun D. Coffey C. S. Drever P. Fourwinds S. (2011). Very early hypothermia induction in patients with severe brain injury (the National Acute Brain Injury Study: Hypothermia II): a randomised trial. Lancet Neurol. 10, 131–139 10.1016/S1474-4422(10)70300-8 21169065 PMC3628679

[B28] Coleshaw S. R. Van Someren R. N. Wolff A. H. Davis H. M. Keatinge W. R. (1983). Impaired memory registration and speed of reasoning caused by low body temperature. J. Appl. Physiol. 55, 27–31 6885583 10.1152/jappl.1983.55.1.27

[B29] Collins C. A. Rojas E. (1982). Temperature dependence of the sodium channel gating kinetics in the node of Ranvier. Q. J. Exp. Physiol. 67, 41–55 6281846 10.1113/expphysiol.1982.sp002623

[B30] Craig A. D. Chen K. Bandy D. Reiman E. M. (2000). Thermosensory activation of insular cortex. Nat. Neurosci. 3, 184–190 10.1038/72131 10649575

[B31] Crone C. (1963). The permeability of capillaries in various organs as determined by use of the ‘indicator diffusion’ method. Acta Physiol. Scand. 58, 292–305 10.1111/j.1748-1716.1963.tb02652.x 14078649

[B32] Cushing H. (1914). Studies on the Cerebro-Spinal Fluid: I. Introduction. J. Med. Res. 31, 1–19 19972189 PMC2094441

[B33] Cypess A. M. Doyle A. N. Sass C. A. Huang T. L. Mowschenson P. M. Rosen H. N. (2013). Quantification of human and rodent brown adipose tissue function using 99mTc-methoxyisobutylisonitrile SPECT/CT and 18F-FDG PET/CT. J. Nucl. Med. 54, 1896–1901 10.2967/jnumed.113.121012 24071505 PMC4012367

[B34] Deja M. A. Malinowski M. Golba K. S. Kajor M. Lebda-Wyborny T. Hudziak D. (2009). Diazoxide protects myocardial mitochondria, metabolism, and function during cardiac surgery: a double-blind randomized feasibility study of diazoxide-supplemented cardioplegia. J. Thorac Cardiovasc. Surg. 137, 997–1004, 1004e1001–1002. 10.1016/j.jtcvs.2008.08.068 19327530

[B35] Delgado J. M. Hanai T. (1966a). Intracerebral temperatures in free-moving cats. Am. J. Physiol. 211, 755–769 5927906 10.1152/ajplegacy.1966.211.3.755

[B36] Delgado J. M. Hanai T. (1966b). Intracerebral temperatures in free-moving cats. Am. J. Physiol. 211, 755–769 5927906 10.1152/ajplegacy.1966.211.3.755

[B37] Dietrich W. D. Atkins C. M. Bramlett H. M. (2009). Protection in animal models of brain and spinal cord injury with mild to moderate hypothermia. J. Neurotrauma 26, 301–312 10.1089/neu.2008.0806 19245308 PMC2848835

[B38] Dietrich W. D. Bramlett H. M. (2010). The evidence for hypothermia as a neuroprotectant in traumatic brain injury. Neurotherapeutics 7, 43–50 10.1016/j.nurt.2009.10.015 20129496 PMC2819078

[B39] Duschek S. Hellmann N. Merzoug K. Reyes Del Paso G. A. Werner N. S. (2012). Cerebral blood flow dynamics during pain processing investigated by functional transcranial Doppler sonography. Pain Med. 13, 419–426 10.1111/j.1526-4637.2012.01329.x 22299810

[B40] Duvernoy H. M. Delon S. Vannson J. L. (1981). Cortical blood vessels of the human brain. Brain Res. Bull. 7, 519–579 10.1016/0361-9230(81)90007-1 7317796

[B41] Ecker R. D. Goerss S. J. Meyer F. B. Cohen-Gadol A. A. Britton J. W. Levine J. A. (2002). Vision of the future: initial experience with intraoperative real-time high-resolution dynamic infrared imaging. Technical note. J. Neurosurg. 97, 1460–1471 10.3171/jns.2002.97.6.1460 12507150

[B42] Elias H. Schwartz D. (1969). Surface areas of the cerebral cortex of mammals determined by stereological methods. Science 166, 111–113 10.1126/science.166.3901.111 4897390

[B43] El-Sawalhi M. M. Ahmed L. A. (2014). Exploring the protective role of apocynin, a specific NADPH oxidase inhibitor, in cisplatin-induced cardiotoxicity in rats. Chem. Biol. Interact. 207, 58–66 10.1016/j.cbi.2013.11.008 24291008

[B44] Erickson C. A. Jung M. W. Mcnaughton B. L. Barnes C. A. (1996). Contribution of single-unit spike waveform changes to temperature-induced alterations in hippocampal population spikes. Exp. Brain Res. 107, 348–360 10.1007/BF00230417 8821377

[B45] Esiri M. M. Gay D. (1990). Immunological and neuropathological significance of the Virchow-Robin space. J. Neurol. Sci. 100, 3–8 10.1016/0022-510X(90)90004-7 2089138

[B46] Feng T. (1936). The heat production of nerve Ergebn. Rev. Physiol. Biochem. Pharmacol. 38, 73–132

[B47] Fingas M. Clark D. L. Colbourne F. (2007). The effects of selective brain hypothermia on intracerebral hemorrhage in rats. Exp. Neurol. 208, 277–284 10.1016/j.expneurol.2007.08.018 17927984

[B48] Fleury C. Neverova M. Collins S. Raimbault S. Champigny O. Levi-Meyrueis C. (1997). Uncoupling protein-2: a novel gene linked to obesity and hyperinsulinemia. Nat. Genet. 15, 269–272 10.1038/ng0397-269 9054939

[B49] Fohlmeister J. F. Cohen E. D. Newman E. A. (2010). Mechanisms and distribution of ion channels in retinal ganglion cells: using temperature as an independent variable. J. Neurophysiol. 103, 1357–1374 10.1152/jn.00123.2009 20053849 PMC2887638

[B50] Fukuda M. Ono T. Nakamura K. (1987). Functional relations among inferotemporal cortex, amygdala, and lateral hypothalamus in monkey operant feeding behavior. J. Neurophysiol. 57, 1060–1077 3585454 10.1152/jn.1987.57.4.1060

[B51] Fuxe K. Rivera A. Jacobsen K. X. Hoistad M. Leo G. Horvath T. L. (2005). Dynamics of volume transmission in the brain. Focus on catecholamine and opioid peptide communication and the role of uncoupling protein 2. J. Neural. Transm. 112, 65–76 10.1007/s00702-004-0158-3 15599605

[B52] Gahwiler B. H. Mamoon A. M. Schlapfer W. T. Tobias C. A. (1972). Effects of temperature on spontaneous bioelectric activity of cultured nerve cells. Brain Res. 40, 527–533 10.1016/0006-8993(72)90157-6 5027177

[B53] Gao Y. Zorman S. Gundersen G. Xi Z. Ma L. Sirinakis G. (2012). Single reconstituted neuronal SNARE complexes zipper in three distinct stages. Science 337, 1340–1343 10.1126/science.1224492 22903523 PMC3677750

[B54] Garbuzova-Davis S. Haller E. Williams S. N. Haim E. D. Tajiri N. Hernandez-Ontiveros D. G. (2014). Compromised blood-brain barrier competence in remote brain areas in ischemic stroke rats at chronic stage. J. Comp. Neurol. 522, 3120–3137 10.1002/cne.23582 24610730 PMC4107178

[B55] Gelman B. Schleien C. L. Lohe A. Kuluz J. W. (1996). Selective brain cooling in infant piglets after cardiac arrest and resuscitation. Crit. Care Med. 24, 1009–1017 10.1097/00003246-199606000-00022 8681567

[B56] George J. M. Jin H. Woods W. S. Clayton D. F. (1995). Characterization of a novel protein regulated during the critical period for song learning in the zebra finch. Neuron 15, 361–372 10.1016/0896-6273(95)90040-3 7646890

[B57] Gheibi S. Aboutaleb N. Khaksari M. Kalalian-Moghaddam H. Vakili A. Asadi Y. (2014). Hydrogen sulfide protects the brain against ischemic reperfusion injury in a transient model of focal cerebral ischemia. J. Mol. Neurosci. 54, 264–270 10.1007/s12031-014-0284-9 24643521

[B58] Girard P. Bullier J. (1989). Visual activity in area V2 during reversible inactivation of area 17 in the macaque monkey. J. Neurophysiol. 62, 1287–1302 2600626 10.1152/jn.1989.62.6.1287

[B59] Gluckman P. D. Wyatt J. S. Azzopardi D. Ballard R. Edwards A. D. Ferriero D. M. (2005). Selective head cooling with mild systemic hypothermia after neonatal encephalopathy: multicentre randomised trial. Lancet 365, 663–670 10.1016/S0140-6736(05)17946-X 15721471

[B60] Gorbach A. M. Heiss J. Kufta C. Sato S. Fedio P. Kammerer W. A. (2003). Intraoperative infrared functional imaging of human brain. Ann. Neurol. 54, 297–309 10.1002/ana.10646 12953262

[B61] Graw J. A. Von Haefen C. Poyraz D. Mobius N. Sifringer M. Spies C. D. (2013). Chronic alcohol consumption increases the expression of uncoupling protein-2 and -4 in the brain. Alcohol. Clin. Exp. Res. 37, 1650–1656 10.1111/acer.12144 23800309

[B62] Greeley W. J. Kern F. H. Ungerleider R. M. Boyd J. L. 3rd. Quill T. Smith L. R. (1991). The effect of hypothermic cardiopulmonary bypass and total circulatory arrest on cerebral metabolism in neonates, infants, and children. J. Thorac. Cardiovasc. Surg. 101, 783–794 2023435

[B63] Green A. R. Ashwood T. (2005). Free radical trapping as a therapeutic approach to neuroprotection in stroke: experimental and clinical studies with NXY-059 and free radical scavengers. Curr. Drug Targets CNS Neurol. Disord. 4, 109–118 10.2174/1568007053544156 15857295

[B64] Greenfield J. G. Love S. Louis D. N. Ellison D. (2008). Greenfield's Neuropathology. London: Hodder Arnold

[B65] Guatteo E. Chung K. K. Bowala T. K. Bernardi G. Mercuri N. B. Lipski J. (2005). Temperature sensitivity of dopaminergic neurons of the substantia nigra pars compacta: involvement of transient receptor potential channels. J. Neurophysiol. 94, 3069–3080 10.1152/jn.00066.2005 16014800

[B66] Guedj E. (2009). Neuroimaging findings in fibromyalgia: what clinical impact? Joint Bone Spine 76, 224–226 10.1016/j.jbspin.2009.01.004 19369105

[B67] Guyton A. C. Hall J. E. (2006). Textbook of Medical Physiology. Philadelphia: Elsevier Saunders

[B68] Hagerdal M. Harp J. Nilsson L. Siesjo B. K. (1975). The effect of induced hypothermia upon oxygen consumption in the rat brain. J. Neurochem. 24, 311–316 10.1111/j.1471-4159.1975.tb11881.x 1113108

[B69] Hajat C. Hajat S. Sharma P. (2000). Effects of poststroke pyrexia on stroke outcome: a meta-analysis of studies in patients. Stroke 31, 410–414 10.1161/01.STR.31.2.410 10657414

[B70] Hales J. R. Fawcett A. A. Bennett J. W. Needham A. D. (1978). Thermal control of blood flow through capillaries and arteriovenous anastomoses in skin of sheep. Pflugers Arch. 378, 55–63 10.1007/BF00581958 569825

[B71] Hamilton C. L. (1963). Hypothalamic temperature records of a monkey. Proc. Soc. Exp. Biol. Med. 112, 55–57 10.3181/00379727-112-27948 13952326

[B72] Hammel H. T. Jackson D. C. Stolwijk J. A. Hardy J. D. Stromme S. B. (1963). Temperature regulation by hypothalamic proportional control with an adjustable set point. J. Appl. Physiol. 18, 1146–1154 14080734 10.1152/jappl.1963.18.6.1146

[B73] Haque N. Ludri A. Hossain S. A. Ashutosh M. (2012). Comparative studies on temperature threshold for heat shock protein 70 induction in young and adult Murrah buffaloes. J. Anim. Physiol. Anim. Nutr. (Berl.) 96, 920–929 10.1111/j.1439-0396.2011.01208.x 21848850

[B74] Haveman J. Sminia P. Wondergem J. Van Der Zee J. Hulshof M. C. (2005). Effects of hyperthermia on the central nervous system: what was learnt from animal studies? Int. J. Hyperthermia 21, 473–487 10.1080/02656730500159079 16048843

[B75] Hayward J. N. Baker M. A. (1968a). Role of cerebral arterial blood in the regulation of brain temperature in the monkey. Am. J. Physiol. 215, 389–403 4969787 10.1152/ajplegacy.1968.215.2.389

[B76] Hayward J. N. Baker M. A. (1968b). Role of cerebral arterial blood in the regulation of brain temperature in the monkey. Am. J. Physiol. 215, 389–403 4969787 10.1152/ajplegacy.1968.215.2.389

[B77] Hayward J. N. Baker M. A. (1969). A comparative study of the role of the cerebral arterial blood in the regulation of brain temperature in five mammals. Brain Res. 16, 417–440 10.1016/0006-8993(69)90236-4 4311724

[B78] He X. (2011). Thermostability of biological systems: fundamentals, challenges, and quantification. Open Biomed. Eng. J. 5, 47–73 10.2174/1874120701105010047 21769301 PMC3137158

[B79] Helmholtz H. F. (1848). Über die Wärmeentwicklung der Muskelaction. Arch. f. Anat. Physiol. 15, 144–164

[B80] Hemingway A. Robinson R. Hemingway C. Wall J. (1966). Cutaneous and brain temperatures related to respiratory metabolism of the sheep. J. Appl. Physiol. 21, 1223–1227 5916654 10.1152/jappl.1966.21.4.1223

[B81] Hernandez-Resendiz S. Buelna-Chontal M. Correa F. Zazueta C. (2013). Targeting mitochondria for cardiac protection. Curr. Drug Targets 14, 586–600 10.2174/1389450111314050008 23458575

[B82] Hill A. V. (1929). The heat production and recovery of crustacean nerve. Proc. R. Soc. Lond. B 105, 133–176 10.1098/rspb.1929.0035

[B83] Hirashima Y. Takaba M. Endo S. Hayashi N. Yamashita K. Takaku A. (1998). Intracerebral temperature in patients with hydrocephalus of varying aetiology. J. Neurol. Neurosurg. Psychiatr. 64, 792–794 10.1136/jnnp.64.6.792 9647313 PMC2170125

[B84] Hodgkin A. L. Huxley A. F. (1952). Movement of sodium and potassium ions during nervous activity. Cold Spring Harb. Symp. Quant. Biol. 17, 43–52 10.1101/SQB.1952.017.01.007 13049154

[B85] Horvath T. L. Warden C. H. Hajos M. Lombardi A. Goglia F. Diano S. (1999). Brain uncoupling protein 2: uncoupled neuronal mitochondria predict thermal synapses in homeostatic centers. J. Neurosci. 19, 10417–10427 10575039 10.1523/JNEUROSCI.19-23-10417.1999 PMC6782406

[B86] Howarth C. Gleeson P. Attwell D. (2012). Updated energy budgets for neural computation in the neocortex and cerebellum. J. Cereb. Blood Flow Metab. 32, 1222–1232 10.1038/jcbfm.2012.35 22434069 PMC3390818

[B87] Hutchings M. Weller R. O. (1986). Anatomical relationships of the pia mater to cerebral blood vessels in man. J. Neurosurg. 65, 316–325 10.3171/jns.1986.65.3.0316 3734882

[B88] Hutchison J. S. Ward R. E. Lacroix J. Hebert P. C. Barnes M. A. Bohn D. J. (2008). Hypothermia therapy after traumatic brain injury in children. N. Engl. J. Med. 358, 2447–2456 10.1056/NEJMoa0706930 18525042

[B89] Huxley A. F. (1957). An ultramicrotome. J. Physiol. 137, 73P–74P 13463786

[B90] Hypothermia after Cardiac Arrest Study Group. (2002). Mild therapeutic hypothermia to improve the neurologic outcome after cardiac arrest. N. Engl. J. Med. 346, 549–556 10.1056/NEJMoa012689 11856793

[B91] Inamura A. Adachi Y. Inoue T. He Y. Tokuda N. Nawata T. (2013). Cooling treatment transiently increases the permeability of brain capillary endothelial cells through translocation of claudin-5. Neurochem. Res. 38, 1641–1647 10.1007/s11064-013-1066-4 23653089

[B92] Jackson D. C. Hammel H. T. (1963). Reduced Set Point Temperature in Exercising Dog. Techn Docum Rep Amrl-Tdr-63-93. AMRL TR. 1–16 14131186

[B93] James W. (1892). The stream of consciousness, in Psychology, Chapter XI (Cleveland, New York: World Publishing Company).

[B94] Jasper H. H. Shacter D. G. Montplaisir J. (1970). The effect of local cooling upon spontaneous and evoked electrical activity of cerebral cortex. Can. J. Physiol. Pharmacol. 48, 640–652 10.1139/y70-094 5479360

[B95] Jiang J. Y. Gao G. Y. Li W. P. Yu M. K. Zhu C. (2002). Early indicators of prognosis in 846 cases of severe traumatic brain injury. J. Neurotrauma 19, 869–874 10.1089/08977150260190456 12184856

[B96] Johanson C. E. Duncan J. A. 3rd. Klinge P. M. Brinker T. Stopa E. G. Silverberg G. D. (2008). Multiplicity of cerebrospinal fluid functions: new challenges in health and disease. Cerebrospinal Fluid Res. 5:10 10.1186/1743-8454-5-10 18479516 PMC2412840

[B97] Kalil R. E. Chase R. (1970). Corticofugal influence on activity of lateral geniculate neurons in the cat. J. Neurophysiol. 33, 459–474 4314735 10.1152/jn.1970.33.3.459

[B98] Kalmbach A. S. Waters J. (2012). Brain surface temperature under a craniotomy. J. Neurophysiol. 108, 3138–3146 10.1152/jn.00557.2012 22972953 PMC3544864

[B99] Katz B. Miledi R. (1965). The effect of temperature on the synaptic delay at the neuromuscular junction. J. Physiol. 181, 656–670 5880384 10.1113/jphysiol.1965.sp007790 PMC1357674

[B100] Kawamura H. Sawyer C. H. (1965a). Elevation in brain temperature during paradoxical sleep. Science 150, 912–913 10.1126/science.150.3698.912 5835794

[B101] Kawamura H. Sawyer C. H. (1965b). Elevation in brain temperature during paradoxical sleep. Science 150, 912–913 10.1126/science.150.3698.912 5835794

[B102] Keating E. G. Gooley S. G. (1988). Saccadic disorders caused by cooling the superior colliculus or the frontal eye field, or from combined lesions of both structures. Brain Res. 438, 247–255 10.1016/0006-8993(88)91343-1 3345431

[B103] Khatri N. Man H. Y. (2013). Synaptic activity and bioenergy homeostasis: implications in brain trauma and neurodegenerative diseases. Front. Neurol. 4:199 10.3389/fneur.2013.00199 24376435 PMC3858785

[B104] Kim F. Nichol G. Maynard C. Hallstrom A. Kudenchuk P. J. Rea T. (2013). Effect of prehospital induction of mild hypothermia on survival and neurological status among adults with cardiac arrest: a randomized clinical trial. JAMA. 311, 45–52 10.1001/jama.2013.282173 24240712 PMC13045629

[B105] King C. Robinson T. Dixon C. E. Rao G. R. Larnard D. Nemoto C. E. (2010). Brain temperature profiles during epidural cooling with the ChillerPad in a monkey model of traumatic brain injury. J. Neurotrauma 27, 1895–1903 10.1089/neu.2009.1178 20684677

[B106] Kiyatkin E. A. (2010). Brain temperature homeostasis: physiological fluctuations and pathological shifts. Front. Biosci. 15, 73–92 10.2741/3608 20036808 PMC3149793

[B107] Kiyatkin E. A. Bae D. (2008). Behavioral and brain temperature responses to salient environmental stimuli and intravenous cocaine in rats: effects of diazepam. Psychopharmacology (Berl.) 196, 343–356 10.1007/s00213-007-0965-y 17938891

[B108] Kiyatkin E. A. Brown P. L. (2004). Modulation of physiological brain hyperthermia by environmental temperature and impaired blood outflow in rats. Physiol. Behav. 83, 467–474 10.1016/j.physbeh.2004.08.032 15581669

[B109] Kiyatkin E. A. Brown P. L. Wise R. A. (2002). Brain temperature fluctuation: a reflection of functional neural activation. Eur. J. Neurosci. 16, 164–168 10.1046/j.1460-9568.2002.02066.x 12153543

[B110] Kiyatkin E. A. Mitchum R. D. Jr. (2003). Fluctuations in brain temperature during sexual interaction in male rats: an approach for evaluating neural activity underlying motivated behavior. Neuroscience 119, 1169–1183 10.1016/S0306-4522(03)00222-7 12831871

[B111] Kiyatkin E. A. Sharma H. S. (2009). Permeability of the blood-brain barrier depends on brain temperature. Neuroscience 161, 926–939 10.1016/j.neuroscience.2009.04.004 19362131 PMC2694729

[B112] Kovalzon V. M. (1973). Brain temperature variations during natural sleep and arousal in white rats. Physiol. Behav. 10, 667–670 10.1016/0031-9384(73)90141-8 4708980

[B113] Kuluz J. W. Prado R. Chang J. Ginsberg M. D. Schleien C. L. Busto R. (1993). Selective brain cooling increases cortical cerebral blood flow in rats. Am. J. Physiol. 265, H824–H827 8214116 10.1152/ajpheart.1993.265.3.H824

[B114] Lamanna J. C. Mccracken K. A. Patil M. Prohaska O. J. (1989). Stimulus-activated changes in brain tissue temperature in the anesthetized rat. Metab. Brain Dis. 4, 225–237 10.1007/BF00999769 2601641

[B115] Lamanna J. C. Rosenthal M. Novack R. Moffett D. F. Jobsis F. F. (1980). Temperature coefficients for the oxidative metabolic responses to electrical stimulation in cerebral cortex. J. Neurochem. 34, 203–209 10.1111/j.1471-4159.1980.tb04641.x 6256472

[B116] Laptook A. R. Shalak L. Corbett R. J. (2001). Differences in brain temperature and cerebral blood flow during selective head versus whole-body cooling. Pediatrics 108, 1103–1110 10.1542/peds.108.5.1103 11694688

[B117] Lassen N. A. (1985). Normal average value of cerebral blood flow in younger adults is 50 ml/100 g/min. J. Cereb. Blood Flow Metab. 5, 347–349 10.1038/jcbfm.1985.48 4030914

[B118] Lee J. C. Callaway J. C. Foehring R. C. (2005). Effects of temperature on calcium transients and Ca^2+^-dependent afterhyperpolarizations in neocortical pyramidal neurons. J. Neurophysiol. 93, 2012–2020 10.1152/jn.01017.2004 15548621

[B119] Li Q. Khatibi N. Zhang J. H. (2014). Vascular neural network: the importance of vein drainage in stroke. Transl. Stroke Res. 5, 163–166 10.1007/s12975-014-0335-0 24563018 PMC3985555

[B120] Lomber S. G. Payne B. R. Cornwell P. (1996). Learning and recall of form discriminations during reversible cooling deactivation of ventral-posterior suprasylvian cortex in the cat. Proc. Natl. Acad. Sci. U.S.A. 93, 1654–1658 10.1073/pnas.93.4.1654 8643686 PMC39997

[B121] Lomber S. G. Payne B. R. Horel J. A. (1999). The cryoloop: an adaptable reversible cooling deactivation method for behavioral or electrophysiological assessment of neural function. J. Neurosci. Methods 86, 179–194 10.1016/S0165-0270(98)00165-4 10065985

[B122] Long M. A. Fee M. S. (2008). Using temperature to analyse temporal dynamics in the songbird motor pathway. Nature 456, 189–194 10.1038/nature07448 19005546 PMC2723166

[B123] Lu L. Zhang C. Cai Q. Lu Q. Duan C. Zhu Y. (2013). Voltage-dependent anion channel involved in the alpha-synuclein-induced dopaminergic neuron toxicity in rats. Acta Biochim. Biophys. Sin. 45, 170–178 10.1093/abbs/gms114 23291291

[B124] Lucchesi B. R. (1990). Myocardial ischemia, reperfusion and free radical injury. Am. J. Cardiol. 65, 14I–23I 10.1016/0002-9149(90)90120-P 1692444

[B125] Lust A. Fuller A. Maloney S. K. Mitchell D. Mitchell G. (2007). Thermoregulation in pronghorn antelope (Antilocapra americana Ord) in the summer. J. Exp. Biol. 210, 2444–2452 10.1242/jeb.005587 17601948

[B126] Mack W. J. Mocco J. Ducruet A. F. Laufer I. King R. G. Zhang Y. (2006). A cerebroprotective dose of intravenous citrate/sorbitol-stabilized dehydroascorbic acid is correlated with increased cerebral ascorbic acid and inhibited lipid peroxidation after murine reperfused stroke. Neurosurgery 59, 383–388 discussion: 383–388. 10.1227/01.NEU.0000223496.96945.A7 16883179

[B127] Madsen P. L. Sperling B. K. Warming T. Schmidt J. F. Secher N. H. Wildschiodtz G. (1993). Middle cerebral artery blood velocity and cerebral blood flow and O2 uptake during dynamic exercise. J. Appl. Physiol. 74, 245–250 8444699 10.1152/jappl.1993.74.1.245

[B128] Maloney S. K. Mitchell D. Mitchell G. Fuller A. (2007). Absence of selective brain cooling in unrestrained baboons exposed to heat. Am. J. Physiol. Regul. Integr. Comp. Physiol. 292, R2059–R2067 10.1152/ajpregu.00809.2006 17218437

[B129] Marklund N. Farrokhnia N. Hanell A. Vanmechelen E. Enblad P. Zetterberg H. (2013). Monitoring of β-amyloid dynamics after human traumatic brain injury. J. Neurotrauma 31, 42–55 10.1089/neu.2013.2964 23829439

[B130] Mascio C. E. Myers J. A. Edmonds H. L. Austin E. H. 3rd. (2009). Near-infrared spectroscopy as a guide for an intermittent cerebral perfusion strategy during neonatal circulatory arrest. ASAIO J. 55, 287–290 10.1097/MAT.0b013e3181964861 19282752

[B131] Masuda H. Hirata A. Kawai H. Wake K. Watanabe S. Arima T. (2011). Local exposure of the rat cortex to radiofrequency electromagnetic fields increases local cerebral blood flow along with temperature. J. Appl. Physiol. 110, 142–148 10.1152/japplphysiol.01035.2010 21030669

[B132] Matthias A. Ohlson K. B. Fredriksson J. M. Jacobsson A. Nedergaard J. Cannon B. (2000). Thermogenic responses in brown fat cells are fully UCP1-dependent. UCP2 or UCP3 do not substitute for UCP1 in adrenergically or fatty scid-induced thermogenesis. J. Biol. Chem. 275, 25073–25081 10.1074/jbc.M000547200 10825155

[B133] Mattson M. P. Kroemer G. (2003). Mitochondria in cell death: novel targets for neuroprotection and cardioprotection. Trends Mol. Med. 9, 196–205 10.1016/S1471-4914(03)00046-7 12763524

[B134] Maybhate A. Hu C. Bazley F. A. Yu Q. Thakor N. V. Kerr C. L. (2012). Potential long-term benefits of acute hypothermia after spinal cord injury: assessments with somatosensory-evoked potentials. Crit. Care Med. 40, 573–579 10.1097/CCM.0b013e318232d97e 22001581 PMC3261348

[B135] Mcelligott J. G. Melzack R. (1967a). Localized thermal changes evoked in the brain by visual and auditory stimulation. Exp. Neurol. 17, 293–312 10.1016/0014-4886(67)90108-2 6019262

[B136] Mcelligott J. G. Melzack R. (1967b). Localized thermal changes evoked in the brain by visual and auditory stimulation. Exp. Neurol. 17, 293–312 10.1016/0014-4886(67)90108-2 6019262

[B137] Mellergard P. (1995). Intracerebral temperature in neurosurgical patients: intracerebral temperature gradients and relationships to consciousness level. Surg. Neurol. 43, 91–95 10.1016/0090-3019(95)80049-M 7701435

[B138] Mellergard P. Nordstrom C. H. (1990). Epidural temperature and possible intracerebral temperature gradients in man. Br. J. Neurosurg. 4, 31–38 10.3109/02688699009000679 2334525

[B139] Michenfelder J. D. Milde J. H. (1991). The relationship among canine brain temperature, metabolism, and function during hypothermia. Anesthesiology 75, 130–136 10.1097/00000542-199107000-00021 2064037

[B140] Milhorat T. H. (1975). The third circulation revisited. J. Neurosurg. 42, 628–645 10.3171/jns.1975.42.6.0628 167134

[B141] Miller G. Stein F. Trevino R. David Y. Contant C. F. Jefferson L. S. (1999). Rectal-scalp temperature difference predicts brain death in children. Pediatr. Neurol. 20, 267–269 10.1016/S0887-8994(98)00146-5 10328274

[B142] Min L. J. Mogi M. Tsukuda K. Jing F. Ohshima K. Nakaoka H. (2014). Direct stimulation of angiotensin II type 2 receptor initiated after stroke ameliorates ischemic brain damage. Am. J. Hypertens. 27, 1036–1044 10.1093/ajh/hpu015 24572705

[B143] Mitchell G. Fuller A. Maloney S. K. Rump N. Mitchell D. (2006). Guttural pouches, brain temperature and exercise in horses. Biol. Lett. 2, 475–477 10.1098/rsbl.2006.0469 17148434 PMC1686210

[B144] Moriyama E. (1990). Cerebral blood flow changes during localized hyperthermia. Neurol. Med. Chir. (Tokyo) 30, 923–929 10.2176/nmc.30.923 1710320

[B145] Moser E. I. Mathiesen L. I. (1996). Relationship between neuronal activity and brain temperature in rats. Neuroreport 7, 1876–1880 10.1097/00001756-199607290-00038 8905684

[B146] Murray A. J. Anderson R. E. Watson G. C. Radda G. K. Clarke K. (2004). Uncoupling proteins in human heart. Lancet 364, 1786–1788 10.1016/S0140-6736(04)17402-3 15541452

[B147] Nakada T. Kwee I. L. Fujii Y. Knight R. T. (2005). High-field, T2 reversed MRI of the hippocampus in transient global amnesia. Neurology 64, 1170–1174 10.1212/01.WNL.0000156158.48587.EA 15824342

[B148] Nakagawa K. Hills N. K. Kamel H. Morabito D. Patel P. V. Manley G. T. (2011). The effect of decompressive hemicraniectomy on brain temperature after severe brain injury. Neurocrit. Care 15, 101–106 10.1007/s12028-010-9446-y 21061187 PMC3627059

[B149] Neumar R. W. Nolan J. P. Adrie C. Aibiki M. Berg R. A. Bottiger B. W. (2008). Post-cardiac arrest syndrome: epidemiology, pathophysiology, treatment, and prognostication. A consensus statement from the International Liaison Committee on Resuscitation (American Heart Association, Australian and New Zealand Council on Resuscitation, European Resuscitation Council, Heart and Stroke Foundation of Canada, InterAmerican Heart Foundation, Resuscitation Council of Asia, and the Resuscitation Council of Southern Africa); the American Heart Association Emergency Cardiovascular Care Committee; the Council on Cardiovascular Surgery and Anesthesia; the Council on Cardiopulmonary, Perioperative, and Critical Care; the Council on Clinical Cardiology; and the Stroke Council. Circulation 118, 2452–2483 10.1161/CIRCULATIONAHA.108.190652 18948368

[B150] Nielsen N. Wetterslev J. Cronberg T. Erlinge D. Gasche Y. Hassager C. (2013). Targeted temperature management at 33 degrees C versus 36 degrees C after cardiac arrest. N. Engl. J. Med. 369, 2197–2206 10.1056/NEJMoa1310519 24237006

[B151] Nybo L. Moller K. Volianitis S. Nielsen B. Secher N. H. (2002a). Effects of hyperthermia on cerebral blood flow and metabolism during prolonged exercise in humans. J. Appl. Physiol. 93, 58–64 10.1152/japplphysiol.00049 12070186

[B152] Nybo L. Nielsen B. (2001). Middle cerebral artery blood velocity is reduced with hyperthermia during prolonged exercise in humans. J. Physiol. 534, 279–286 10.1111/j.1469-7793.2001.t01-1-00279.x 11433008 PMC2278686

[B153] Nybo L. Secher N. H. Nielsen B. (2002b). Inadequate heat release from the human brain during prolonged exercise with hyperthermia. J. Physiol. 545, 697–704 10.1113/jphysiol.2002.030023 12456844 PMC2290690

[B154] O'Collins V. E. Macleod M. R. Donnan G. A. Horky L. L. Van Der Worp B. H. Howells D. W. (2006). 1,026 experimental treatments in acute stroke. Ann. Neurol. 59, 467–477 10.1002/ana.20741 16453316

[B155] Ogawa S. Lee T. M. Nayak A. S. Glynn P. (1990). Oxygenation-sensitive contrast in magnetic resonance image of rodent brain at high magnetic fields. Magn. Reson. Med. 14, 68–78 10.1002/mrm.1910140108 2161986

[B156] Ogawa S. Menon R. S. Tank D. W. Kim S. G. Merkle H. Ellermann J. M. (1993). Functional brain mapping by blood oxygenation level-dependent contrast magnetic resonance imaging. A comparison of signal characteristics with a biophysical model. Biophys. J. 64, 803–812 10.1016/S0006-3495(93)81441-3 8386018 PMC1262394

[B157] Ohmoto Y. Fujisawa H. Ishikawa T. Koizumi H. Matsuda T. Ito H. (1996). Sequential changes in cerebral blood flow, early neuropathological consequences and blood-brain barrier disruption following radiofrequency-induced localized hyperthermia in the rat. Int. J. Hyperthermia 12, 321–334 10.3109/02656739609022521 9044902

[B158] Oku T. Fujii M. Tanaka N. Imoto H. Uchiyama J. Oka F. (2009). The influence of focal brain cooling on neurophysiopathology: validation for clinical application. J. Neurosurg. 110, 1209–1217 10.3171/2009.1.JNS08499 19284241

[B159] Paulson O. B. Hasselbalch S. G. Rostrup E. Knudsen G. M. Pelligrino D. (2010). Cerebral blood flow response to functional activation. J. Cereb. Blood Flow Metab. 30, 2–14 10.1038/jcbfm.2009.188 19738630 PMC2872188

[B160] Percy A. Widman S. Rizzo J. A. Tranquilli M. Elefteriades J. A. (2009). Deep hypothermic circulatory arrest in patients with high cognitive needs: full preservation of cognitive abilities. Ann. Thorac. Surg. 87, 117–123 10.1016/j.athoracsur.2008.10.025 19101283

[B161] Raichle M. E. (1983). Neurogenic control of blood-brain barrier permeability. Acta Neuropathol. Suppl. 8, 75–79 10.1007/978-3-642-68970-3_6 6575566

[B162] Raichle M. E. Mintun M. A. (2006). Brain work and brain imaging. Annu. Rev. Neurosci. 29, 449–476 10.1146/annurev.neuro.29.051605.112819 16776593

[B163] Rajanikant G. K. Zemke D. Senut M. C. Frenkel M. B. Chen A. F. Gupta R. (2007). Carnosine is neuroprotective against permanent focal cerebral ischemia in mice. Stroke 38, 3023–3031 10.1161/STROKEAHA.107.488502 17916766

[B164] Rampone A. J. Shirasu M. E. (1964). Temperature changes in the rat in response to feeding. Science 144, 317–319 10.1126/science.144.3616.317 14169727

[B165] Redzic Z. B. Preston J. E. Duncan J. A. Chodobski A. Szmydynger-Chodobska J. (2005). The choroid plexus-cerebrospinal fluid system: from development to aging. Curr. Top. Dev. Biol. 71, 1–52 10.1016/S0070-2153(05)71001-2 16344101

[B166] Reite M. L. Pegram G. V. (1968). Cortical temperature during paradoxical sleep in the monkey. Electroencephalogr. Clin. Neurophysiol. 25, 36–41 10.1016/0013-4694(68)90084-9 4174781

[B167] Rennels M. L. Blaumanis O. R. Grady P. A. (1990). Rapid solute transport throughout the brain via paravascular fluid pathways. Adv. Neurol. 52, 431–439 2396537

[B168] Ribrault C. Sekimoto K. Triller A. (2011). From the stochasticity of molecular processes to the variability of synaptic transmission. Nat. Rev. Neurosci. 12, 375–387 10.1038/nrn3025 21685931

[B169] Richard D. Rivest R. Huang Q. Bouillaud F. Sanchis D. Champigny O. (1998). Distribution of the uncoupling protein 2 mRNA in the mouse brain. J. Comp. Neurol. 397, 549–560 9699915

[B170] Ritchie J. M. (1973). Energetic aspects of nerve conduction: the relationships between heat production, electrical activity and metabolism. Prog. Biophys. Mol. Biol. 26, 147–187 10.1016/0079-6107(73)90019-9 4145345

[B171] Rittenberger J. C. Callaway C. W. (2013). Temperature management and modern post-cardiac arrest care. N. Engl. J. Med. 369, 2262–2263 10.1056/NEJMe1312700 24237007

[B172] Robinson A. D. Ramanathan K. B. Mcgee J. E. Newman K. P. Weber K. T. (2011). Oxidative stress and cardiomyocyte necrosis with elevated serum troponins: pathophysiologic mechanisms. Am. J. Med. Sci. 342, 129–134 10.1097/MAJ.0b013e3182231ee3 21747281

[B173] Rosen A. D. (2001). Nonlinear temperature modulation of sodium channel kinetics in GH(3) cells. Biochim. Biophys. Acta 1511, 391–396 10.1016/S0005-2736(01)00301-7 11286982

[B174] Rossi S. Zanier E. R. Mauri I. Columbo A. Stocchetti N. (2001). Brain temperature, body core temperature, and intracranial pressure in acute cerebral damage. J. Neurol. Neurosurg. Psychiatr. 71, 448–454 10.1136/jnnp.71.4.448 11561026 PMC1763520

[B175] Rost B. R. Nicholson P. Ahnert-Hilger G. Rummel A. Rosenmund C. Breustedt J. (2011). Activation of metabotropic GABA receptors increases the energy barrier for vesicle fusion. J. Cell Sci. 124, 3066–3073 10.1242/jcs.074963 21852427

[B176] Sabbah H. N. Tocchetti C. G. Wang M. Daya S. Gupta R. C. Tunin R. S. (2013). Nitroxyl (HNO): a novel approach for the acute treatment of heart failure. Circ. Heart Fail. 6, 1250–1258 10.1161/CIRCHEARTFAILURE.113.000632 24107588 PMC4110196

[B177] Sachdev R. N. Ebner F. F. Wilson C. J. (2004). Effect of subthreshold up and down states on the whisker-evoked response in somatosensory cortex. J. Neurophysiol. 92, 3511–3521 10.1152/jn.00347.2004 15254074

[B178] Saltin B. Gagge A. P. Bergh U. Stolwijk J. A. (1972). Body temperatures and sweating during exhaustive exercise. J. Appl. Physiol. 32, 635–643 5038852 10.1152/jappl.1972.32.5.635

[B179] Schiller P. H. Stryker M. Cynader M. Berman N. (1974). Response characteristics of single cells in the monkey superior colliculus following ablation or cooling of visual cortex. J. Neurophysiol. 37, 181–194 4204566 10.1152/jn.1974.37.1.181

[B180] Schlessinger M. Spiro I. J. (1995). Infrared Technology Fundamentals. New York, NY: M. Dekker

[B181] Schmielau F. Singer W. (1977). The role of visual cortex for binocular interactions in the cat lateral geniculate nucleus. Brain Res. 120, 354–361 10.1016/0006-8993(77)90914-3 832128

[B182] Schwartz A. E. Stone J. G. Finck A. D. Sandhu A. A. Mongero L. B. Adams D. C. (1996). Isolated cerebral hypothermia by single carotid artery perfusion of extracorporeally cooled blood in baboons. Neurosurgery 39, 577–581 discussion: 581–572. 8875489 10.1097/00006123-199609000-00028

[B183] Sedunova E. V. (1992). [Brain temperature in small birds and mammals]. Fiziol. Zh. SSSR Im. I M Sechenova 78, 85–89 1330722

[B184] Segal M. B. (1993). Extracellular and cerebrospinal fluids. J. Inherit. Metab. Dis. 16, 617–638 10.1007/BF00711896 8412010

[B185] Serota H. (1939a). Temperature changes in the cortex and hypothalamus during sleep. J. Neurophysiol. 2, 42–47

[B186] Serota H. G. (1938). Localized temperature changes in the cat brain. J. Neurophysiol. 1, 115–124

[B187] Serota H. M. (1939b). Temperature changes in the cortex and hypothalamus during sleep. J. Neurophysiol. 2, 42–47

[B188] Serota H. M. Gerard R. W. (1938). Localized temperature changes in the cat brain. J. Neurophysiol. 1, 115–124

[B189] Serpell L. C. Berriman J. Jakes R. Goedert M. Crowther R. A. (2000). Fiber diffraction of synthetic alpha-synuclein filaments shows amyloid-like cross-beta conformation. Proc. Natl. Acad. Sci. U.S.A. 97, 4897–4902 10.1073/pnas.97.9.4897 10781096 PMC18329

[B190] Shankaran S. Laptook A. R. Ehrenkranz R. A. Tyson J. E. Mcdonald S. A. Donovan E. F. (2005). Whole-body hypothermia for neonates with hypoxic-ischemic encephalopathy. N. Engl. J. Med. 353, 1574–1584 10.1056/NEJMcps050929 16221780

[B191] Sharma H. S. Hoopes P. J. (2003). Hyperthermia induced pathophysiology of the central nervous system. Int. J. Hyperthermia 19, 325–354 10.1080/0265673021000054621 12745974

[B192] Sharma S. Aramburo A. Rafikov R. Sun X. Kumar S. Oishi P. E. (2013). L-carnitine preserves endothelial function in a lamb model of increased pulmonary blood flow. Pediatr. Res. 74, 39–47 10.1038/pr.2013.71 23628882 PMC3709010

[B193] Shevelev I. A. (1998). Functional imaging of the brain by infrared radiation (thermoencephaloscopy). Prog. Neurobiol. 56, 269–305 10.1016/S0301-0082(98)00038-0 9770241

[B194] Siesjö B. K. (1978). Brain Energy Metabolism. Chichester; New York, NY: Wiley

[B195] Simoens P. Lauwers H. De Geest J. P. De Schaepdrijver L. (1987). Functional morphology of the cranial retia mirabilia in the domestic mammals. Schweiz. Arch. Tierheilkd. 129, 295–307 3616596

[B196] Smith C. M. Adelson P. D. Chang Y. F. Brown S. D. Kochanek P. M. Clark R. S. (2011). Brain-systemic temperature gradient is temperature-dependent in children with severe traumatic brain injury. Pediatr. Crit. Care Med. 12, 449–454 10.1097/PCC.0b013e3181f390dd 20711083 PMC5055080

[B197] Soukup J. Zauner A. Doppenberg E. M. Menzel M. Gilman C. Young H. F. (2002). The importance of brain temperature in patients after severe head injury: relationship to intracranial pressure, cerebral perfusion pressure, cerebral blood flow, and outcome. J. Neurotrauma 19, 559–571 10.1089/089771502753754046 12042092

[B198] Squire L. R. (2012). Fundamental Neuroscience. Amsterdam; Boston: Elsevier

[B199] Stone J. G. Goodman R. R. Baker K. Z. Baker C. J. Solomon R. A. (1997). Direct intraoperative measurement of human brain temperature. Neurosurgery 41, 20–24 10.1097/00006123-199707000-00007 9218291

[B200] Stoodley M. A. Brown S. A. Brown C. J. Jones N. R. (1997). Arterial pulsation-dependent perivascular cerebrospinal fluid flow into the central canal in the sheep spinal cord. J. Neurosurg. 86, 686–693 10.3171/jns.1997.86.4.0686 9120633

[B201] Suehiro E. Fujisawa H. Koizumi H. Nomura S. Kajiwara K. Fujii M. (2011). Significance of differences between brain temperature and core temperature (delta T) during mild hypothermia in patients with diffuse axonal injury. Neurol. Med. Chir. (Tokyo) 51, 551–555 10.2176/nmc.51.551 21869574

[B202] Sullivan R. M. Wilson D. A. Leon M. (1988). Physical stimulation reduces the brain temperature of infant rats. Dev. Psychobiol. 21, 237–250 10.1002/dev.420210305 3371556 PMC1892163

[B203] Sundgren P. C. Petrou M. Harris R. E. Fan X. Foerster B. Mehrotra N. (2007). Diffusion-weighted and diffusion tensor imaging in fibromyalgia patients: a prospective study of whole brain diffusivity, apparent diffusion coefficient, and fraction anisotropy in different regions of the brain and correlation with symptom severity. Acad. Radiol. 14, 839–846 10.1016/j.acra.2007.03.015 17574134

[B204] Swan H. (1974). Thermoregulation and Bioenergetics; Patterns for Vertebrate Survival. New York, NY: American Elsevier Pub. Co

[B205] Taketomo T. Saito A. (1965). Experimental studies on cerebrospinal fluid flow. Neurology 15, 578–586 10.1212/WNL.15.6.578 14312781

[B206] Thompson S. M. Masukawa L. M. Prince D. A. (1985). Temperature dependence of intrinsic membrane properties and synaptic potentials in hippocampal CA1 neurons *in vitro*. J. Neurosci. 5, 817–824 3973697 10.1523/JNEUROSCI.05-03-00817.1985 PMC6565032

[B207] Thornton K. (2003). The electrophysiological effects of a brain injury on auditory memory functioning. The QEEG correlates of impaired memory. Arch. Clin. Neuropsychol. 18, 363–378 10.1093/arclin/18.4.363 14591452

[B208] Triller A. Choquet D. (2008). New concepts in synaptic biology derived from single-molecule imaging. Neuron 59, 359–374 10.1016/j.neuron.2008.06.022 18701063

[B209] Trimble W. S. Cowan D. M. Scheller R. H. (1988). VAMP-1: a synaptic vesicle-associated integral membrane protein. Proc. Natl. Acad. Sci. U.S.A. 85, 4538–4542 10.1073/pnas.85.12.4538 3380805 PMC280466

[B210] Trubel H. K. Sacolick L. I. Hyder F. (2006). Regional temperature changes in the brain during somatosensory stimulation. J. Cereb. Blood Flow Metab. 26, 68–78 10.1038/sj.jcbfm.9600164 15959461

[B211] Tryba A. K. Ramirez J. M. (2004). Hyperthermia modulates respiratory pacemaker bursting properties. J. Neurophysiol. 92, 2844–2852 10.1152/jn.00752.2003 15190095

[B212] Volgushev M. Vidyasagar T. R. Chistiakova M. Eysel U. T. (2000). Synaptic transmission in the neocortex during reversible cooling. Neuroscience 98, 9–22 10.1016/S0306-4522(00)00109-3 10858607

[B213] Wagner H. J. Pilgrim C. Brandl J. (1974). Penetration and removal of horseradish peroxidase injected into the cerebrospinal fluid: role of cerebral perivascular spaces, endothelium and microglia. Acta Neuropathol. 27, 299–315 10.1007/BF00690695 4366438

[B214] Walter B. Bauer R. Kuhnen G. Fritz H. Zwiener U. (2000). Coupling of cerebral blood flow and oxygen metabolism in infant pigs during selective brain hypothermia. J. Cereb. Blood Flow Metab. 20, 1215–1224 10.1097/00004647-200008000-00007 10950382

[B215] Wang G. Hamid T. Keith R. J. Zhou G. Partridge C. R. Xiang X. (2010). Cardioprotective and antiapoptotic effects of heme oxygenase-1 in the failing heart. Circulation 121, 1912–1925 10.1161/CIRCULATIONAHA.109.905471 20404253 PMC2917269

[B216] Wang H. Olivero W. Lanzino G. Elkins W. Rose J. Honings D. (2004). Rapid and selective cerebral hypothermia achieved using a cooling helmet. J. Neurosurg. 100, 272–277 10.3171/jns.2004.100.2.0272 15086235

[B217] Wang Q. Tompkins K. D. Simonyi A. Korthuis R. J. Sun A. Y. Sun G. Y. (2006a). Apocynin protects against global cerebral ischemia-reperfusion-induced oxidative stress and injury in the gerbil hippocampus. Brain Res. 1090, 182–189 10.1016/j.brainres.2006.03.060 16650838

[B218] Wang Y. Kimura K. Inokuma K. Saito M. Kontani Y. Kobayashi Y. (2006b). Potential contribution of vasoconstriction to suppression of heat loss and homeothermic regulation in UCP1-deficient mice. Pflugers Arch. 452, 363–369 10.1007/s00424-005-0036-3 16395600

[B219] Weimer M. S. Hanke W. (2005). Correlation between the durations of refractory period and intrinsic optical signal of retinal spreading depression during temperature variations. Exp. Brain Res. 161, 201–208 10.1007/s00221-004-2060-5 15502987

[B220] Whitby J. D. Dunkin L. J. (1971). Cerebral, oesophageal and nasopharyngeal temperatures. Br. J. Anaesth. 43, 673–676 10.1093/bja/43.7.673 5564234

[B221] Wolfson L. I. Katzman R. Escriva A. (1974). Clearance of amine metabolites from the cerebrospinal fluid: the brain as a “sink.” Neurology 24, 772–779 10.1212/WNL.24.8.772 4858421

[B222] Xie T. Mccann U. D. Kim S. Yuan J. Ricaurte G. A. (2000). Effect of temperature on dopamine transporter function and intracellular accumulation of methamphetamine: implications for methamphetamine-induced dopaminergic neurotoxicity. J. Neurosci. 20, 7838–7845 11027249 10.1523/JNEUROSCI.20-20-07838.2000 PMC6772867

[B223] Yablonskiy D. A. Ackerman J. J. Raichle M. E. (2000). Coupling between changes in human brain temperature and oxidative metabolism during prolonged visual stimulation. Proc. Natl. Acad. Sci. U.S.A. 97, 7603–7608 10.1073/pnas.97.13.7603 10861022 PMC16592

[B224] Yang X. F. Chang J. H. Rothman S. M. (2003). Long-lasting anticonvulsant effect of focal cooling on experimental neocortical seizures. Epilepsia 44, 1500–1505 10.1111/j.0013-9580.2003.23003.x 14636319

[B225] Yu Y. Hill A. P. Mccormick D. A. (2012). Warm body temperature facilitates energy efficient cortical action potentials. PLoS Comput. Biol. 8:e1002456 10.1371/journal.pcbi.1002456 22511855 PMC3325181

[B226] Yusuf J. Khan M. U. Cheema Y. Bhattacharya S. K. Weber K. T. (2012). Disturbances in calcium metabolism and cardiomyocyte necrosis: the role of calcitropic hormones. Prog. Cardiovasc. Dis. 55, 77–86 10.1016/j.pcad.2012.02.004 22824113 PMC3404408

[B227] Zenker W. Kubik S. (1996). Brain cooling in humans–anatomical considerations. Anat. Embryol. (Berl.) 193, 1–13 10.1007/BF00186829 8838492

[B228] Zhang E. T. Inman C. B. Weller R. O. (1990). Interrelationships of the pia mater and the perivascular (Virchow-Robin) spaces in the human cerebrum. J. Anat. 170, 111–123 2254158 PMC1257067

[B229] Zhou J. Empey P. E. Bies R. R. Kochanek P. M. Poloyac S. M. (2011). Cardiac arrest and therapeutic hypothermia decrease isoform-specific cytochrome P450 drug metabolism. Drug Metab. Dispos. 39, 2209–2218 10.1124/dmd.111.040642 21868471 PMC3226379

[B230] Zhu M. Ackerman J. J. Yablonskiy D. A. (2009). Body and brain temperature coupling: the critical role of cerebral blood flow. J. Comp. Physiol. B 179, 701–710 10.1007/s00360-009-0352-6 19277681 PMC2843754

